# The language network is not engaged in object categorization

**DOI:** 10.1093/cercor/bhad289

**Published:** 2023-08-09

**Authors:** Yael Benn, Anna A Ivanova, Oliver Clark, Zachary Mineroff, Chloe Seikus, Jack Santos Silva, Rosemary Varley, Evelina Fedorenko

**Affiliations:** Department of Psychology, Manchester Metropolitan University, Manchester M15 6BH, United Kingdom; Brain and Cognitive Sciences Department, Massachusetts Institute of Technology, Cambridge, MA 02139, United States; McGovern Institute for Brain Research, Massachusetts Institute of Technology, Cambridge, MA 02139, United States; Department of Psychology, Manchester Metropolitan University, Manchester M15 6BH, United Kingdom; Brain and Cognitive Sciences Department, Massachusetts Institute of Technology, Cambridge, MA 02139, United States; McGovern Institute for Brain Research, Massachusetts Institute of Technology, Cambridge, MA 02139, United States; Division of Psychology & Language Sciences, University College London, London WC1E 6BT, UK; Division of Psychology & Language Sciences, University College London, London WC1E 6BT, UK; Division of Psychology & Language Sciences, University College London, London WC1E 6BT, UK; Brain and Cognitive Sciences Department, Massachusetts Institute of Technology, Cambridge, MA 02139, United States; McGovern Institute for Brain Research, Massachusetts Institute of Technology, Cambridge, MA 02139, United States

**Keywords:** aphasia, categorization, fMRI, language

## Abstract

The relationship between language and thought is the subject of long-standing debate. One claim states that language facilitates categorization of objects based on a certain feature (e.g. color) through the use of category labels that reduce interference from other, irrelevant features. Therefore, language impairment is expected to affect categorization of items grouped by a single feature (low-dimensional categories, e.g. “Yellow Things”) more than categorization of items that share many features (high-dimensional categories, e.g. “Animals”). To test this account, we conducted two behavioral studies with individuals with aphasia and an fMRI experiment with healthy adults. The aphasia studies showed that selective low-dimensional categorization impairment was present in some, but not all, individuals with severe anomia and was not characteristic of aphasia in general. fMRI results revealed little activity in language-responsive brain regions during both low- and high-dimensional categorization; instead, categorization recruited the domain-general multiple-demand network (involved in wide-ranging cognitive tasks). Combined, results demonstrate that the language system is not implicated in object categorization. Instead, selective low-dimensional categorization impairment might be caused by damage to brain regions responsible for cognitive control. Our work adds to the growing evidence of the dissociation between the language system and many cognitive tasks in adults.

## Introduction

The role of language in mediating or augmenting thought is the subject of long-standing debate. According to one view, language is necessary for many cognitive functions, such as math, logic, and propositional thought (Darwin 1871; Dennett 1994; Bickerton 1995; Carruthers 2002; Bermúdez 2007; Baldo et al. 2010; Baldo et al. 2015, and others). However, a large body of evidence supports a different view: that language is cognitively and neurally independent from the rest of human cognition. This evidence includes the lack of activity in the language brain regions during non-linguistic tasks (Monti et al. 2009; Fedorenko et al. 2011; Monti et al. 2012; Amalric and Dehaene 2016; Amalric and Dehaene 2019; Ivanova et al. 2021), the retained ability of some individuals with aphasia to perform such tasks (e.g. Varley et al. 2005; Siegal and Varley 2006; Bek et al. 2013; Benn et al. 2013), and variability across cultures in the use of language resources during thought (Kim 2002). However, the role of language is still contested for one important aspect of human cognition: categorization.

Like other animals, humans can convert rich, multi-dimensional perceptual inputs into a latent lower-dimensional structured representation of the world. Grouping discriminable individual objects and events into classes allows us not only to decide whether some new object/event belongs to a particular category, but also to draw powerful inferences about shared properties from one category member to another (Mervis and Rosch 1981; Smith and Medin 1981; Wasserman et al. 1988; Smith and Heise 1992; Pearce 1994; Mareschal and Quinn 2001; Murphy 2002).

In contrast to other animals, humans additionally label individual categories with words—the core building blocks of a powerful communication system that allows us to share complex thoughts with one another. Even though categorization is a basic cognitive capacity that evolved long before language, evidence exists that word learning affects category learning in development (e.g. Gershkoff-Stowe et al. 1997; Sloutsky and Fisher 2004; Plunkett et al. 2008; Waxman and Gelman 2009; Ferguson and Waxman 2017) and, to some extent, in adulthood (Lupyan et al. 2007; Brojde et al. 2011; Lupyan and Casasanto 2015). Here, we ask the following: how does language affect the process of grouping objects into categories when the category boundaries are already known?

### High-dimensional and low-dimensional categories

Before summarizing the key prior evidence, it is important to introduce a distinction that some have considered to be relevant to the question of whether language affects categorization. Lupyan and colleagues (e.g. Lupyan 2009; Lupyan and Mirman 2013; Perry and Lupyan 2014) distinguish between “high-dimensional” (HD) categories, where members share many features, and “low-dimensional” (LD) categories, where members share one or a few features. HD categories typically correspond to established sets that reflect either the taxonomic (similarity-based) or relational/thematic (co-occurrence-based) structure of the world (Bain 1855; Mirman et al. 2017). Taxonomic HD categories can often be labeled by superordinate terms such as ANIMALS, FRUIT, or TOOLS. Relational HD categories correspond to common events/scenarios: for example, THINGS YOU TAKE ON A PICNIC or NON-FOOD THINGS FOUND IN THE KITCHEN. For such relational categories, the shared features have to do with typical co-occurrences (e.g. although a fridge and a spatula are quite different, they both co-occur with a large number of kitchen objects, like a stove, pots and pans, a kettle, etc.). In contrast to HD categories, LD categories are more likely to be novel groupings of items that often straddle taxonomic and relational boundaries, such as THINGS MADE OF WOOD or THINGS THAT ARE YELLOW (e.g. things made of wood may include a cupboard, a sledge, and a wooden spoon, and things that are yellow may include a lemon, a yellow hat, and a canary).

Similar distinctions have been made by others, in related literatures. For example, Barsalou (1983) distinguishes between “common” categories, which mirror the correlational structure of the environment, and “ad-hoc,” or “goal-derived,” categories, which are constructed for a specific goal and are thus often based on a small number of features. Kloos and Sloutsky (2008) and Sloutsky (2010) distinguish between “dense” and “sparse” categories based on the ratio of category-relevant variance to total variance. Members of statistically dense categories share many inter-correlated features that matter for category membership, and members of sparse categories have very few features in common, with many other features varying independently and being irrelevant for category membership. Couchman et al. (2010) contrast family-resemblance categorization, which relies on judgments of overall similarity, considering multiple features in tandem, and criterial-attribute categorization (or “rule-based categorization”), which requires adhering to a single-dimensional criterial attribute and suppressing all other, irrelevant dimensions (see also Ashby and O’Brien 2005). Langland et al. (2021) relate the HD/LD distinction to the concrete/abstract distinction, arguing that items in concrete categories have many shared features, whereas identifying items from an abstract category requires generalizing over many irrelevant properties to identify a small set of commonalities. In this work, we use the HD/LD category distinction proposed by Lupyan et al. (although see the discussion for criticisms of that distinction).

### The LD-specific language recruitment hypothesis

One claim that emerged in the literature in recent years is that language plays a special role in LD categorization (Lupyan 2009; Lupyan 2012; Lupyan and Mirman 2013). The argument goes as follows: during LD categorization, only one to two features are relevant to the task, whereas the rest of the features interfere and have to be inhibited; for instance, when categorizing objects by color, their shape and function have to be ignored. A verbal label (e.g. “yellow”) can help maintain focus on the relevant categorization criterion and reduce interference from irrelevant features. The hypothesis states that language resources are used to maintain the label and are therefore more important for LD categorization compared with holistic, HD categorization.

The LD-specific language recruitment hypothesis predicts that reduced availability of language resources should lead to a greater disruption of LD compared with HD categorization.

**Fig. 1:**
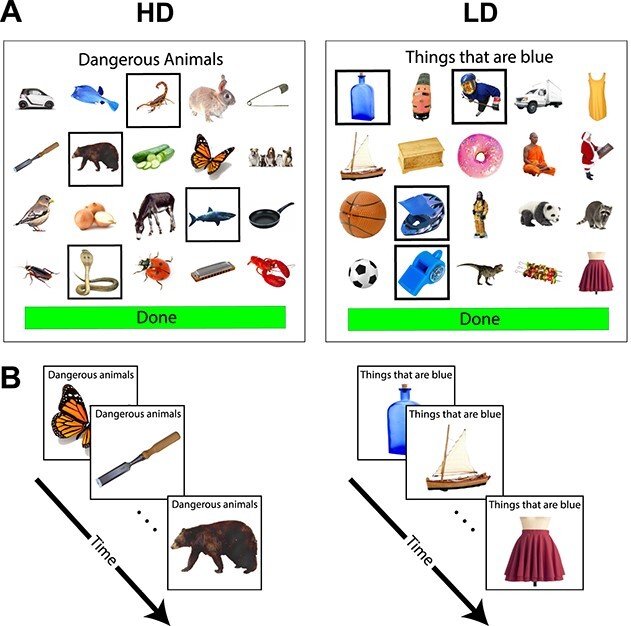
Trial structure in (A) Aphasia Study 1 and (B) Aphasia Study 2 and the fMRI experiment. HD, high dimensional category; LD, low dimensional category.

This prediction found some support in the aphasia literature. Some patients with linguistic deficits have been reported to exhibit impairments in non-verbal categorization tasks when the task required focusing on one particular dimension and ignoring other salient dimensions (De Renzi and Spinnler 1967; Cohen et al. 1980; Cohen and Woll 1981; Hjelmquist 1989; Davidoff and Roberson 2004). Building on these findings, Lupyan (2009) manipulated verbal versus spatial interference in a dual-task paradigm in neurotypical participants and found that verbal, but not visuo-spatial, interference affected the participants’ ability to decide whether an object belongs to an LD category. In contrast, verbal and visuo-spatial interference had similar (and negligible) effects on HD categorization. In a follow-up study, Lupyan and Mirman (2013) directly compared performance on HD and LD categorization in individuals with aphasia and neurotypical controls. Participants were provided with a category descriptor (or label) and then had to select from a picture array the subset of objects that belong to the target category (similar to Fig. 1, top). Performance in the LD condition was lower for both groups, but critically, the HD versus LD difference was larger in individuals with aphasia, particularly in those with low scores on a picture-naming task. Lupyan and colleagues therefore concluded that access to lexical resources is important for LD categorization.

However, evidence from aphasia does not provide uniform support for the LD-specific language recruitment hypothesis. For example, Burger and Muma (1980) found deficits in HD categorization in individuals with anomia and in individuals with Wernicke’s aphasia using a task similar to that used in Lupyan and Mirman (2013). Others described aphasia-related categorization deficits for both HD and LD categories (Koemeda-Lutz et al. 1987) or no deficits in either (Hough 1993). Further, variations in the task (such as showing the category label to the participant during the entire trial versus just at the beginning of the trial) significantly affected categorization performance in participants with aphasia (Koemeda-Lutz et al. 1987), suggesting that task demands may contribute to the observed results (above and beyond alleged effects of category type). Finally, some have argued for a relationship between categorization difficulties and conceptual-semantic rather than purely linguistic impairments (Caramazza et al. 1982; Whitehouse et al. 1978; cf. Le Dorze and Nespoulous 1989).

### The possible role of cognitive control mechanisms in LD categorization

Even if individuals with aphasia consistently showed a selective impairment in LD categorization, this result would not necessarily implicate language as the source of the deficit. In particular, the language network in the left hemisphere, especially in the left frontal cortex, lies adjacent to the domain-general multiple demand network, which supports executive functions, like working memory (WM) and inhibitory control (Duncan 2010; Fedorenko et al. 2012; Duncan 2013; Fedorenko et al. 2013; Assem et al. 2020b). As a result, left hemisphere damage can lead to joint linguistic and domain-general executive deficits (Gainotti et al. 1986; Baldo et al. 2010). Prior work has shown that performance on executive function tasks, not language tasks, predicts success in learning novel categories (Vallila-Rohter and Kiran 2015), and LD categorization consist of novel grouping of elements that are not typically grouped together. Further, the multiple demand network, but not the language network, is robustly sensitive to cognitive effort across domains (e.g. Fedorenko et al. 2011; Fedorenko et al. 2013; Hugdahl et al. 2015; Shashidhara et al. 2019), and LD categorization appears to be more cognitively challenging than HD categorization: LD categories are harder to learn for both human children (e.g. Kloos and Sloutsky 2008) and non-human primates (Couchman et al. 2010), require supervision (e.g. Kloos and Sloutsky 2008), and are generally linked with executively-taxing intentional learning (Kemler Nelson 1984; Ashby et al. 1998; Ashby and Ell 2001; Ashby and O’Brien 2005; Couchman et al. 2010). It is therefore possible that impaired performance on LD categorization (and on categorization tasks more broadly) depends on domain-general cognitive control resources rather than on language resources.

The LD-specific language recruitment hypothesis further predicts that LD categories would evoke stronger activity within the language brain regions. To our knowledge, this hypothesis has not been directly tested in the neuroimaging literature; instead, many studies have investigated differences between taxonomic and thematic relations (Sachs et al. 2008; Kalénine et al. 2009; Sass et al. 2009; Lewis et al. 2015), both of which are considered HD. Further, few neuroimaging studies employ methods that would be required to dissociate the contributions of language-specific regions from those of domain-general cognitive control regions: given the inter-individual variability in the precise locations of functional areas, voxels in anatomically identical locations within the frontal lobe might be language-specific in one individual and domain-general in another, so traditional group-based analyses (Friston et al. 1994) would fail to distinguish between them (Fedorenko et al. 2012; Nieto-Castañón and Fedorenko 2012; Fedorenko and Blank 2020). Disentangling the role of language and executive resources in LD categorization requires identification of language-specific and domain-general cognitive control regions in individual participants and testing their responses to LD compared with HD conditions.

### Current study

Here, we re-examine the role of language in LD and HD categorization by reporting evidence from two behavioral studies with patients with aphasia (and patients with Parkinson’s disease and healthy adults as controls) and an functional Magnetic Resonance Imaging (fMRI) study. In Study 1, we use the setup from Lupyan and Mirman (2013; L&M henceforth) to determine whether their findings can be replicated in a sample of participants with moderate aphasia. In Study 2, we adjust the experimental paradigm to reduce task complexity by decreasing the amount of visual information on the screen at any one time, and test whether the LD-selective categorization impairment holds in a sample of individuals with severe anomia. In the fMRI study, we collect data from neurotypical individuals to test the prediction that the language system is engaged during LD categorization more than during HD categorization.

To foreshadow our results, the LD-selective categorization impairment was observed only in some participants with severe anomia (Study 2), not in the general aphasia sample (Study 1). Only three of the five individuals with severe anomia exhibited an LD-selective categorization impairment, casting doubt at the immediate causal link between language (or naming ability) and LD categorization. Finally, the fMRI study revealed low engagement of the language network during both LD and HD categorization, with no significant difference between the two. Thus, the influence of language on LD categorization is behaviorally inconsistent and is not supported by fMRI evidence, leading us to conclude that the language system does not play a special role in LD (single-feature-based) categorization and is not engaged during categorization in general.

## Aphasia Study 1

The aim of Study 1 was to test the LD-specific language recruitment hypothesis using a paradigm that is closely related to the original L&M study. L&M compared LD and HD categorization performance in participants with anomic aphasia and in neurotypical controls. They found (i) lower performance on LD compared with HD categories in both healthy adults and participants with anomic aphasia; and, critically, (ii) a greater decrement in performance for the LD, compared with the HD condition in participants with aphasia. We explored whether these same effects would replicate in our sample of participants with moderate aphasia. To additionally examine the extent to which performance might depend on the general effect of brain damage, as opposed to a linguistic impairment, we also included a group of individuals with Parkinson’s disease (PD).

### Methods

#### Participants

Neurotypical older participants (*n* = 9 (6 F), age *M* = 67.89, *SD* = 14.98) were recruited by convenience sampling; individuals with chronic aphasia (*n* = 11 (3 F), age *M* = 61.18, *SD* = 12.09) were recruited from the UCL Aphasia Clinic Research Register. The aphasia group included patients with a range of aphasia types and severities. Unlike L&M, we did not try to limit our sample to individuals classified as having “Anomic” aphasia, given that the use of such rigid classification labels fails to account for the heterogeneity among the symptoms observed across patients (Badecker and Caramazza 1985; Caramazza and Badecker 1989; Wilson et al. 2023), and given that some degree of anomia is present in all forms of aphasia (e.g. Goodglass and Geschwind 1976; Blumstein 1988). According to the normative literature on the Boston Naming Test (BNT; Goodglass et al. 1983), which recommends accounting for age, education, and gender when diagnosing anomia (Welch et al. 1996; Zec et al. 2007), 7 of the 11 participants in the aphasia group (P1, P4, P6, P8, P9, P10, and P11) were below the cut-off for normative naming performance. Individuals with PD (*n* = 13 (8 F), age *M* = 68.08, *SD* = 8.20) were recruited from the Parkinson’s UK Research Registry. For detailed participant information, see Table 1. All participants used English as their primary language. Patients were offered a £10.00 reimbursement. Ethical approval was granted by the UCL Research Ethics panel, Project ID: LC/2013/05, and all volunteers gave informed consent to participate in the study.

**Table 1:** Participant information, study 1.

Group	Participant	Age	Education	Gender	TPO (months)	BNT	HD accuracy (SD)	LD accuracy (SD)
Neurotypical	1	75	Up to 16	F	-	51	97% (17)	96% (20)
	2	68	Up to 16	F	-	55	98% (15)	97% (17)
	3	68	Up to 16	M	-	55	98% (14)	97% (17)
	4	56	Degree-Level	F	-	59	100% (7)	98% (14)
	5	98	Up to 16	F	-	47	94% (24)	93% (26)
	6	54	Degree-Level	M	-	53	99% (11)	97% (17)
	7	69	Up to 16	M	-	55	98% (14)	97% (17)
	8	76	Up to 16	F	-	52	96% (20)	94% (24)
	9	47	Up to 18	F	-	58	99% (12)	97% (17)
PD	1	60	Postgraduate	M	36	59	99% (9)	98% (14)
	2	58	Degree-Level	M	12	58	99% (9)	99% (11)
	3	80	Up to 18	F	48	58	98% (14)	99% (9)
	4	56	Postgraduate	F	48	54	99% (10)	98% (16)
	5	66	Degree-Level	F	72	59	99% (9)	97% (17)
	6	75	Degree-Level	F	96	56	98% (15)	97% (17)
	7	59	Degree-Level	F	60	55	98% (16)	97% (18)
	8	69	Postgraduate	F	36	54	100% (7)	96% (19)
	9	63	Postgraduate	F	60	56	98% (14)	98% (16)
	10	77	Degree-Level	M	12	46	98% (15)	99% (9)
	11	72	Postgraduate	M	120	53	96% (19)	98% (14)
	12	75	Degree-Level	M	2	58	98% (15)	96% (19)
	13	75	Postgraduate	F	360	53	96% (20)	96% (20)
Aphasia	1	52	Degree-Level	M	120	30	92% (27)	89% (32)
	2	57	Up to 16	M	84	57	99% (10)	98% (14)
	3	52	Up to 18	M	48	52	98% (15)	97% (18)
	4	59	Postgraduate	M	120	43	100% (7)	98% (15)
	5	79	Up to 16	F	36	50	95% (23)	96% 20)
	6	44	Up to 18	F	12	14	98% (16)	95% (21)
	7	81	Up to 16	M	96	57	92% (27)	95% (21)
	8	56	Up to 18	M	60	12	90% (31)	89% (31)
	9	57	Up to 18	M	48	51	100% (7)	98% (14)
	10	60	Up to 16	M	132	34	96% (19)	95% (22)
	11	76	Up to 16	F	84	14	93% (26)	93% (26)

TPO, time post onset; BNT, Boston Naming Test; HD, high dimension categories; LD, low dimension categories; SD, standard deviation

#### Design and materials

The critical categorization task was modeled closely on L&M’s study, with two modifications. First, the original study used 34 unique categories (18 HD categories and 16 LD categories), with some repetition of categories in each condition. We chose to not repeat any categories, so we limited the materials to 16 categories in each condition (dropping “BODY PARTS” and “FACIAL FEATURES” from the HD set). And second, we used a different set of images. L&M used normed color drawings (Rossion and Pourtois 2004), and we used high-quality color photographs selected from the Hemera Photo Objects 5000 and Google Images. For each category, we selected 8–15 targets and 25–27 distractors. Distractors included some items which were related to the target category (for example, for the category “DANGEROUS ANIMALS,” 13 of the 26 distractors were animals that were not dangerous, and the category “ANIMALS WITH STRIPES” included distractors that were animals without stripes, and inanimate objects with stripes). A total of 1087 unique images were used (any given image appeared as a target in 0–2 categories and as a distractor in 0–2 categories). All photographs depicted objects on a white background. The materials and the experimental scripts for all studies are available on OSF: https://osf.io/guwh8/.

To determine the extent of lexical impairment in the aphasia group and to compare lexical abilities across the three groups, all participants completed the BNT (Goodglass et al. 1983), where they were sequentially presented with up to 60 line drawings of objects and asked to overtly name each one. The standard discontinuation rule was applied, with testing stopped after eight consecutive failed naming attempts. No semantic or phonological cues were given.

#### Experimental procedure

Testing was carried out individually either in a quiet well-lit room at the UCL Aphasia clinic or at the participants’ home, using a MacBook Pro (Retina, 13-inch display) and an external computer mouse. The study was set up using PsychoPy (Version 1.83), and the procedure closely followed that used in L&M’s study, except where noted. On each trial (see Fig. 1A for a sample HD and LD trial), participants were presented with a 4 x 5 grid of images. The image sets for the individual trials—each consisting of 20 images (4 targets and 16 distractors)—were randomly selected from the pool of targets/distractors for each participant separately. The category was stated at the top of the screen in lower-case Arial bold letters (e.g. “objects that hold water”) and remained on the screen for the duration of the trial. Participants selected the objects that belonged to the target category by clicking on each relevant image. A gray frame appeared around an image once it was clicked; clicking the image again de-selected it (removed the gray frame) to allow participants to modify responses. Once the participant had selected all of the images they deemed appropriate for the target category, they clicked a large green button with the word “Done” at the bottom of the screen (in the L&M version, the button said “click here when done”). Doing so triggered the next trial. Although each trial contained a fixed number of targets (four), participants were not informed of the number of targets during the instructions and could therefore select as many images as they wished on any given trial. No time limit was imposed on the trials, but participants were encouraged to work as quickly and accurately as possible. HD and LD trials were interleaved, and the order of conditions was randomized for each participant. Each participant performed the experiment twice for a total of 64 trials (32 per condition), but in contrast to L&M, different sets of images were used for the two instances of each category to minimize practice effects. Responses were recorded for each image; response times were recorded for each trial (the time from the onset of the trial until the “Done” button was pressed). The session lasted approximately 1 hour. The BNT (Goodglass et al. 2001) was administered between the two runs of the study.

We wish to note that in their study, L&M state that they only included ‘the correct responses’ in their RT analyses. It is not clear what is meant here given the internal complexity of the trials (i.e., possible errors including misses and false alarms). It is possible that L&M only included trials where no errors of any kind were made, but they also talk about ‘per click’ RTs, which is not consistent with this interpretation. It also appears that L&M analyzed median, not mean RTs. For simplicity and to avoid collider bias (Elwert and Winship 2014), we chose to analyze all trials here. We use mean per-trial values, but we make the per-image data available on OSF (https://osf.io/guwh8/), so other researchers could perform additional analyses.

#### Statistical analyses

To determine possible differences in demographics and BNT scores across groups, we conducted ANOVA tests (with follow-up Bonferroni-corrected *t*-tests), implemented in SPSS 22 (IBM Corp 2013). For the critical analyses, we used linear/logistic mixed effect regression models (Baayen et al. 2008). Given that correct or incorrect selection of items is categorical in nature, we use logistic regression to analyze accuracy measures (Jaeger 2008). For response times, we use linear regression. When specifying model contrasts, we used sum coding for category dimension (HD vs. LD); the effect of group was therefore estimated across both category dimensions. For the participant group (neurotypical vs. aphasia vs. PD), we used dummy coding with “neurotypical” as the reference level; thus, the effect of category was estimated specifically for the neurotypical group (with interaction terms denoting whether the category effect differed for the aphasia/PD groups). For completeness and to facilitate result comparison with L&M, we also ran pairwise comparisons across groups using “aphasia” as the reference level (the results were Bonferroni-corrected, *n* = 2). The mixed effect analyses were run using the *lmer* function from the *lme4* R package (Bates et al. 2015); statistical significance of the effects was evaluated using the *lmerTest* package (Kuznetsova et al. 2017); follow-up comparisons were conducted using the *emmeans* package (https://cran.r-project.org/package=emmeans). Lastly, due to a technical error, if participants accidently double-clicked the “Done” button, the next set of images was skipped, and the software registered it as though no response was made by participants. As a result, we excluded trials where no selection was made and where the trial length was less than 5 seconds. This resulted in the exclusion of 40 trials (out of 2,112; ~ 2%), spread randomly between participants, groups and categories. The analysis code is available on OSF: https://osf.io/guwh8/.

### Results

#### Group profiles

As expected, the neurotypical, aphasia, and PD groups differed significantly in their BNT scores (*F*(2,31) = 9.85, *P* < 0.001). Post-hoc pairwise comparisons showed that the BNT scores of participants with aphasia (*M* = 37.64, *SD* = 17.78) were significantly lower than those of neurotypical participants (*P* = 0.005) or participants with PD (*P* = 0.001), with the latter two groups not differing significantly (*M* = 53.89, *SD* = 3.66 vs. *M* = 55.21, *SD* = 3.42; *P* > 0.999). The groups did not differ in age (*F*(2,31) = 1.45, *P* = 0.250), but a significant difference was observed in the level of education (*F*(2,31) = 14.36, *P* < 0.001): participants in the PD group were significantly more educated than both neurotypical participants (*P* = 0.001) and participants with aphasia (*P* = 0.002), with the latter two not differing significantly (*P* > 0.999).

#### Categorization task

**Fig. 2:**
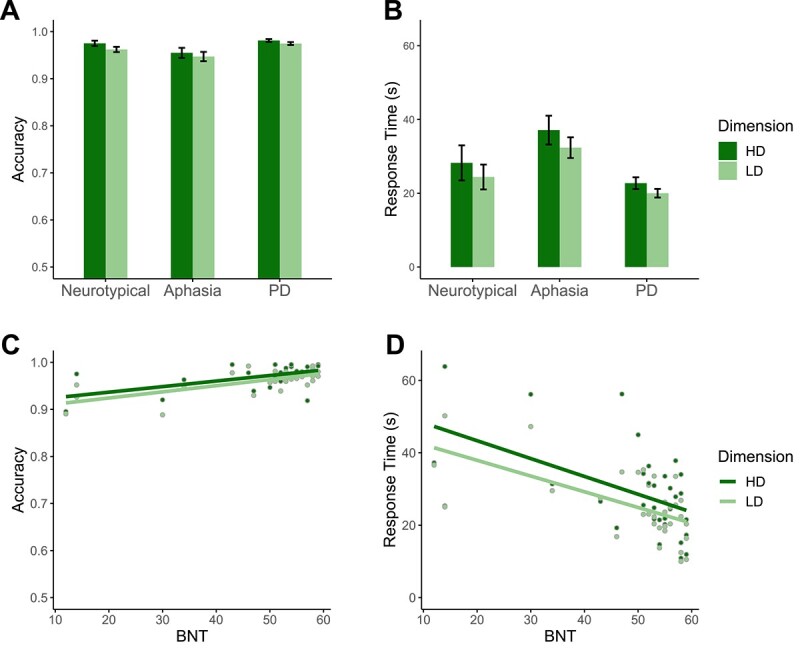
Study 1 results. (A) accuracy and (B) response time (RT) across the three participant groups (here, RT is the time from trial onset until participants pressed the “done” button). (C) Accuracy and (D) RT plotted against participants’ BNT scores, a measure of naming performance. Here and elsewhere, error bars depict the standard error across participants.

Categorization results for Study 1 are summarized in Fig. 2.

##### Accuracy

We did not observe predicted categorization deficits in the aphasia group. Participants with aphasia had high accuracy for both LD (*M* = 0.95, *SD* = 0.03) and HD categories (*M* = 0.95, *SD* = 0.03; LD > HD: β = −0.11, *SE* = 0.26, *P* = 0.672). The overall accuracy for participants with aphasia (*M* = 0.95, *SD* = 0.03) was similar to neurotypical participants (*M* = 0.97, *SD* = 0.02; neurotypical>aphasia: β = 0.36, *SE* = 0.26, *P* = 0.166) and slightly lower than for participants with PD (*M* = 0.98, *SD* = 0.01; PD > aphasia: β = 0.69, *SE* = 0.24, *P* = 0.004). The key comparison—interaction between category dimension and group (aphasia vs. neurotypical)—was marginally significant (β = −0.26, *SE* = 0.14, *P* = 0.055), and the trend was in the opposite direction from that predicted by the LD-specific language recruitment hypothesis (the performance gap for the neurotypical group was larger). The category dimension by group interaction for the aphasia versus PD comparison was not significant (β = −0.12, *SE* = 0.13, *P* = 0.341). Thus, we did not observe LD-specific categorization impairment in the aphasia group.

We additionally conducted an exploratory analysis to investigate the difference between the aphasia and PD groups. Given that the PD group had a higher average education level, we repeated the analysis above with “education level” as an additional fixed effect. The updated model had a similar fit to the data compared with the original (as per the likelihood ratio test: χ^2^ = 1.55, *P* = 0.213); under this model, the difference between the aphasia and the PD groups was no longer significant (β = 0.44, *SE* = 0.31, *P* = 0.158). The significance of other effects was unchanged.

##### Response times

The RT analysis revealed that participants with aphasia were faster to respond during LD trials (*M* = 32.36, *SD* = 9.33) compared with HD trials (*M* = 37.10, *SD* = 12.90; LD > HD: β = −4.75, *SE* = 2.26, *P* = 0.042), in contrast to the predictions of the LD-specific language recruitment hypothesis. The overall RTs for participants with aphasia (*M* = 34.70, *SD* = 11.30) were longer than for neurotypical participants (*M* = 26.30, *SD* = 12.10; β = −8.42, *SE* = 4.02, *P* = 0.044) and the PD group (*M* = 21.40, *SD* = 5.14; β = −13.30, *SE* = 3.66, *P* < 0.001). The interactions between category dimension and group were not significant (neurotypical>aphasia: β = 0.82, *SE* = 1.29, *P* = 0.522; PD > aphasia: β = 1.98, *SE* = 1.17, *P* = 0.091). Follow-up analyses showed no overall effect of category dimension across groups (β = 3.81, *SE* = 2.19, *P* = 0.249), within the neurotypical group (β = 3.92, *SE* = 2.34, *P* = 0.271) or within the PD group (β = 2.76, *SE* = 2.27, *P* = 0.521).

##### Effect of naming performance

To explore the effect of naming ability on the categorization task performance, we fitted a logistic mixed effect linear regression model with the BNT score, category dimension, and their interaction as fixed effects and participants (across the three groups) and categories (e.g. “DANGEROUS ANIMALS”) as random effects. Similar to L&M, we also included education level as a fixed effect.

We found that BNT was a significant predictor of accuracy (β = 0.36, *SE* = 0.08, *P* < 0.001) and RT (β = −5.26, *SE* = 1.41, *P* < 0.001), such that higher BNT scores corresponded to more accurate and faster performance (Fig. 2C, D). There was no main effect of category dimension (accuracy: β = −0.24, *SE* = 0.26, *P* = 0.358; RT: β = −3.73, *SE* = 2.14, *P* = 0.092) and no interaction between BNT and category dimension (accuracy: β = −0.05, *SE* = 0.04, *P* = 0.271; RT: β = 0.74, *SE* = 0.49, *P* = 0.131). Education was a significant predictor for both accuracy (β = 0.23, *SE* = 0.07, *P* = 0.001) and RT (β = −2.95, *SE* = 1.24, *P* = 0.024). Whereas these results indicate that there exists a relationship between the BNT score and categorization performance, they do not support the LD-specific language recruitment hypothesis.

### Interim discussion

In Study 1, we use the setup from a previous study (Lupyan and Mirman 2013, or L&M) to test the hypothesis that language is selectively recruited to support LD categorization. To examine the generality of the language-categorization link, we recruited a group of individuals with aphasia with diverse degrees of aphasia severity. We found that the aphasia group performed comparably to the control groups on the categorization tasks. Naming ability (as measured with the BNT) predicted overall categorization performance, but we observed no interaction between naming ability and category dimension (HD vs LD). In summary, Study 1 provides no support for the hypothesis that language plays a special role in LD categorization.

Participants with aphasia performed object categorization as accurately as the neurotypical controls. Participants with PD performed better than the other groups, but this difference is likely explained by the higher education level in this group. As in L&M, participants with aphasia were significantly slower to complete the categorization task compared with the neurotypical group, and to our additional, PD control group. However, this slower performance in the aphasia group can be explained by the presence of motor impairments (e.g. right hemiplegia)—often more severe than in participants with PD—which often necessitate use of their non-preferred hand. This difference could also be explained by the fact that participants with aphasia may require longer to process the category descriptions, which are presented verbally and sometimes in lengthy phrases (e.g. “NON-FOOD THINGS FOUND IN THE KITCHEN”). Thus, we are hesitant to place a lot of weight on the RT differences.

Across groups, BNT scores significantly predicted performance on all three outcome measures (although this effect did not differ for LD and HD categorization). Although BNT scores may be a proxy for the severity of linguistic impairment, they also might index the degree of executive function impairments (Higby et al. 2019). Due to the proximity of language-specific and multiple-demand brain regions in some parts of the brain (Fedorenko et al. 2012; Fedorenko and Blank 2020), brain damage that causes lower BNT scores is also likely to lead to difficulties with cognitively demanding tasks. The categorization task adopted from L&M involves visual search and selecting among multiple options, which require a substantial degree of cognitive control (Posner and Petersen 1990; Petersen and Posner 2012); thus, categorization difficulties on the current task might reflect this increased recruitment of executive/cognitive control resources.

Given the heterogeneity of the aphasia group in Study 1 and a relatively low sample size, our results in this section should be interpreted with caution. Therefore, in the next two studies, we (i) test the hypothesis that LD-specific categorization impairments might be observed specifically in participants with low BNT scores (Aphasia Study 2) and (ii) evaluate the relative contributions of language and executive resource to categorization in neurotypical participants (fMRI experiment).

For Aphasia Study 2 and the fMRI experiment, we use a modified paradigm that temporally separates the process of reading the category label and the process of categorizing objects based on that label. Reading the label necessarily requires the use of language but is not the target of the LD-specific categorization hypothesis: thus, in the new setup, participants first read the label and then make categorization judgments.

## Aphasia Study 2

The aim of Study 2 was 3-fold. First, we wanted to further probe the relationship between naming ability (BNT scores) and categorization performance, which was reported by L&M and found in Study 1. Thus, we recruited participants with aphasia who had severe anomia, as measured by the BNT (score range 1–11, compared with 12–57 in Study 1; see Tables 1 and 2). Second, we adjusted the paradigm to minimize executive demands, including attention, visual search, selection/inhibition, and updating. Third, we sought to validate a version of the task that could be used in an fMRI setting (time-locked to events). See Fig. 1B for the modified task setup.

**Table 2:** Participant information, study 2.

Group	Participant	Age	Education	Gender	TPO (months)	BNT	HD Accuracy (SD)	LD Accuracy (SD)
Neurotypical	1	68	Degree-Level	F	-	51	99% (11)	98% (14)
	2	61	Postgraduate	F	-	41	98% (12)	98% (12)
	3	85	Degree-Level	F	-	54	99% (10)	95% (21)
	4	73	Postgraduate	F	-	58	95% (21)	96% (19)
	5	72	Up to 18	F	-	58	99% (9)	99% (9)
	6	77	Postgraduate	F	-	55	96% (21)	99% (11)
	7	77	Degree-Level	F	-	59	97% (17)	98% (12)
	8	66	Degree-Level	F	-	57	98% (13)	99% (7)
	9	66	Postgraduate	F	-	54	98% (12)	98% (12)
	10	76	Degree-Level	F	-	59	99% (9)	98% (14)
	11	65	Postgraduate	F	-	56	98% (15)	98% (13)
	12	80	Up to 18	F	-	45	97% (18)	99% (11)
	13	74	Postgraduate	F	-	54	98% (12)	99% (10)
	14	71	Degree-Level	F	-	57	96% (20)	95% (23)
	15	76	Degree-Level	F	-	47	96% (19)	95% (22)
PD	1	71	Degree-Level	M	24	58	98% (13)	97% (17)
	2	78	Degree-Level	M	24	47	95% (22)	93% (25)
	3	64	Postgraduate	M	30	48	98% (13)	95% (21)
	4	72	Postgraduate	M	18	59	98% (14)	96% (19)
	5	54	Degree-Level	M	204	58	97% (16)	97% (16)
	6	72	Degree-Level	M	4	48	96% (21)	98% (14)
	7	62	Postgraduate	F	120	56	98% (14)	99% (9)
	8	65	Postgraduate	M	17	59	97% (17)	98% (13)
	9	74	Up to 18	M	96	56	96% (19)	97% (17)
	10	67	Up to 16	M	60	54	99% (9)	98% (13)
	11	67	Postgraduate	M	72	59	98% (14)	96% (19)
	12	59	Postgraduate	M	30	58	99% (10)	99% (7)
	13	59	Degree-Level	M	48	60	97% (18)	99% (11)
	14	67	Degree-Level	M	18	55	97% (17)	95% (22)
	15	68	Degree-Level	M	98	48	92% (27)	94% (23)
Aphasia	1	58	Up to 18	M	42	5	88% (32)	82% (39)
	2	68	Up to 16	M	68	9	77% (42)	79% (41)
	3	77	Up to 18	M	111	11	91% (29)	88% (33)
	4	57	Degree-Level	M	34	1	96% (19)	95% (22)
	5	73	Up to 18	M	326	4	96% (19)	91% (28)

TPO, time post onset; BNT, Boston Naming Test; HD, high dimension categories; LD, low dimension categories; SD, standard deviation

### Method

#### Participants

Neurotypical participants (*n* = 15 (15 F), age *M* = 72.47, *SD* = 6.41) were recruited by convenience sampling; patients with chronic aphasia and severe lexical impairment (*n* = 5 (all males), age *M* = 66.60, *SD* = 8.91) were recruited from Aphasia volunteer research registers; PD patients (*n* = 15 (1 F), age *M* = 66.60, *SD* = 6.38) were recruited from the Parkinson’s UK Research Registry (see Table 2 for detailed participant information). None of the participants took part in Study 1. All participants used English as their primary language and were offered a £15.00 reimbursement. Ethical approval was granted by the UCL Research Ethics panel, Project ID: LC/2013/05, and all volunteers gave informed consent to participate in the study.

#### Design and materials

The categories were identical to those of Study 1. The images were also largely the same although some were replaced by better quality photographs. Unlike Study 1, we presented the images sequentially (Fig. 2B). Each block started with a category label, followed by 12 images presented one at a time. The category label remained on the screen to minimize memory demands. The images for each category block were randomly selected from the general set of pictures for that category. The number of targets varied across blocks (minimum: 4, maximum: 6) so as to minimize the implicit learning of a fixed number of targets. Categories were grouped by dimension (LD/HD) into groups of four, for a total of eight blocks (four blocks per dimension). These 8-block sequences (“runs”) were separated by a rest period of fixation (10 s in duration). The order of runs, the order of conditions within runs (LD first vs. HD first), the order of categories within runs, and the order of images within category blocks were randomized for each participant.

#### Experimental procedure

Testing was carried out individually either in a quiet well-lit room at a clinic nearest to the participant’s location or in their home, using a Dell Latitude E5540 (14.1-inch display). The paradigm was set up using Python (version 2.7.10). Each category block started with an instruction screen presented for 2 s that read “Please find [CATEGORY LABEL]” (e.g. “Please find objects that hold water”). Given that the participants in the aphasia group had severe lexical impairment and had difficulty processing orthographic information, the experimenter read the category label aloud to all participants (in all groups) during this trial-initial 2 s window. This screen was followed by a sequence of 12 images presented one at a time for a maximum of 10 s per image. For each image, participants had to decide whether the depicted object belong to the target category by pressing one of two keys on the keyboard: the “Y” key marked with a green sticker for YES, or the “N” key marked with a red sticker for NO. If no response was recorded for 10s, the experiment advanced to the next image. Responses and response times were recorded for each image. The session lasted approximately 1 hour. The BNT was administered at the beginning of the testing session.

#### Statistical analyses

The statistical analysis procedure was the same as in Study 1. No trials were excluded.

## Results

### Group profiles

As expected, the groups differed significantly in their BNT scores (*F*(2,32) = 202.67, *P* < 0.001). Post-hoc pairwise comparisons revealed that the BNT scores of participants with aphasia (*M* = 6.00, *SD* = 4.00) were significantly lower than both neurotypical participants (*P* < 0.001) and participants with PD (*P* < 0.001), with the latter two groups not differing significantly (*M* = 53.67, *SD* = 5.42 vs. *M* = 54.87, *SD* = 4.73, *P >* 0.999). The groups did not differ in age (*F*(2,32) = 3.23, *P* = 0.053), but a significant difference was observed in the level of education (*F*(2,32) = 5.42, *P* = 0.009), with neurotypical participants and participants with PD having significantly more years of education than participants with aphasia (*P =* 0.010 and 0.016, respectively). The neurotypical participants and participants with PD did not differ (*P* > 0.999).

#### Categorization task

**Fig. 3:**
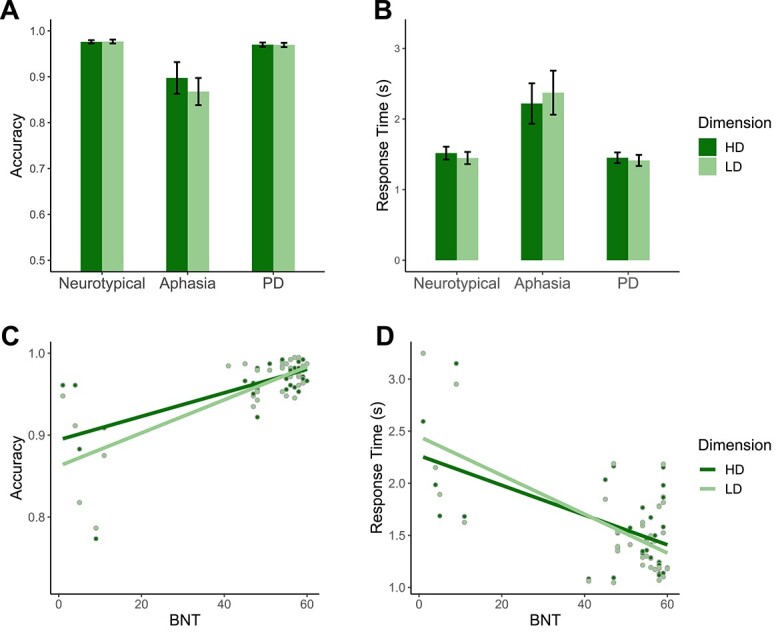
Study 2 results. (A) Accuracy and (B) RT across the three participant groups (here, RT is the time until participants pressed a “yes” or “no” button for each image within a trial). (C) Accuracy and (D) RT plotted against participants’ BNT scores, a measure of naming performance.

Categorization results for Study 2 are summarized in Fig. 3.

##### Accuracy

As in Study 1, participants with aphasia had similar accuracies for LD (*M* = 0.87, *SD* = 0.07) and HD categories (*M* = 0.90, *SD* = 0.08; LD > HD: β = −0.24, *SE* = 0.22, *P* = 0.282). Participants with aphasia had overall lower accuracies (*M* = 0.88, *SD* = 0.07) compared with neurotypical participants (*M* = 0.98, *SD* = 0.01; neurotypical>aphasia: β = 1.70, *SE* = 0.28, *P* < 0.001) and participants with PD (*M* = 0.97, *SD* = 0.02; PD > aphasia: β = 1.44, *SE* = 0.28, *P* < 0.001), which is consistent with the negative relationship between naming ability and categorization performance observed in Study 1. We did not observe a reliable category dimension by group interaction for the aphasia versus neurotypical comparison (β = 0.44, *SE* = 0.26, *P* = 0.086), nor for the aphasia versus PD comparison (β = 0.42, *SE* = 0.23, *P* = 0.070). Critically, in accordance with the LD-specific language recruitment hypothesis, we observed a category dimension by group interaction both for the aphasia versus neurotypical comparison (β = 0.37, *SE* = 0.16, *P* = 0.021) and for the aphasia versus PD comparison (β = 0.32, *SE* = 0.15, *P* = 0.037).

##### Response times

RT results were also consistent with the LD-specific language recruitment hypothesis. Participants with aphasia were slower to respond during LD trials (*M* = 2.37, *SD* = 0.70) compared with HD trials (*M* = 2.22, *SD* = 0.64; LD > HD: β = 0.16, *SE* = 0.08, *P* = 0.044). The overall RTs for participants with aphasia (*M =* 2.30, *SD =* 0.64) were longer than for neurotypical participants (*M =* 1.48, *SD =* 0.34; β = −.81, SE = 0.19, *P* < 0.001) and participants with PD (*M =* 1.43, *SD =* 0.29; β = −0.86, *SE* = 0.19, *P* < 0.001). We also observed an interaction between group and category dimension for both the neurotypical versus aphasia comparison (β = −0.23, *SE* = 0.03, *P* < 0.001) and the PD versus aphasia comparison (β = −0.19, *SE* = 0.03, *P* < 0.001), such that participants with aphasia had longer RTs for LD categories compared with HD categories.

##### Effect of naming performance

As in Study 1, BNT was a significant predictor of categorization performance (accuracy: β = 0.50, *SE* = 0.11, *P* < 0.001; RT: β = −0.29, *SE* = 0.07, *P* < 0.001). There was no main effect of category dimension (accuracy: β = 0.06, *SE* = 0.21, *P* = 0.787; RT: β = −0.02, *SE* = 0.07, *P* = 0.742); however, unlike Study 1, and as predicted by the LD-specific language recruitment hypothesis, we observed an interaction between BNT and category dimension for accuracy (β = 0.13, *SE* = 0.05, *P* = 0.007) and RT (β = −0.08, *SE* = 0.01, *P* < 0.001). Finally, education was not a significant predictor of performance in this dataset (accuracy: β = 0.12, *SE* = 0.13, *P* = 0.372; RT: β = −0.01, *SE* = 0.08, *P* = 0.940).

##### Single case analysis

Although the effect of naming performance in Study 2 is in line with L&M’s prediction, careful examination of individual participants’ scores casts doubt on the causal relationship between naming ability and categorization performance. Specifically, participants A4 and A5 in the aphasia group (Table 2) had very low BNT scores (1/60 and 4/60), but nonetheless performed well relative to both the neurotypical and PD groups (accuracy: LD A4 = 95%; A5 = 91%; HD A4 = 96%; A5 = 96%). Using the Adjusted F Calculator for comparing single cases to groups (Hulleman and Humphreys 2007), these two participants did not differ significantly from the combined neurotypical and PD groups for either the HD condition (A4: *F*[1,29] < 0.01*, P (one-tailed)* = 0.414; A5: *F*[1,29] < 0.01*, P (one-tailed)* = 0.414) or the LD condition (A4: *F*[1,29] = 0.02, *P (one-tailed)* = 0.337; A5: *F*[1,29] = 0.14, *P (one-tailed)* = 0.154). This dissociation indicates that naming impairment is not necessarily accompanied by a decrement in LD categorization.

### Interim discussion

In Study 2, we examined object categorization performance of individuals with severe anomia using a modified task paradigm (with the goal of reducing executive demands). We found that, in accordance with the LD-selective language recruitment hypothesis, individuals with aphasia were impaired on LD categorization more than on HD categorization. However, performance of individual participants offers a reason to be skeptical about a direct link between naming and categorization. Participants A4 and A5 demonstrated dissociation between these two tasks: despite very low BNT scores (lower than 5/60), they performed similarly on the HD and LD categorization trials, and their accuracy on both conditions was well within the range of the control groups.

Dissociations observed in individual case studies are critical in informing debates about cognitive architecture (e.g. Caramazza and McCloskey 1988; Badecker et al. 1991; Caramazza and Coltheart 2006). Naturally occurring brain lesions do not respect the boundaries between functionally distinct brain areas, and comorbidities or associations of impairments are common (e.g. Bates et al. 2003). For example, damage to the left inferior frontal gyrus (LIFG) is likely to cause multiple cognitive impairments due to the high functional heterogeneity of that region (Fedorenko et al. 2012; Fedorenko and Blank 2020). Thus, a correlation that we observe between naming and categorization might be because the brain regions that support these functions are located nearby and thus are likely to be damaged together (rather than naming and categorization engaging the same brain region/mechanism). The dissociation that we observe in participants A4 and A5 supports this possibility: in both cases, severely limited lexical access did not prevent success on the categorization task, revealing that intact linguistic (naming) skills are not necessary for object categorization.

As in Study 1, naming ability significantly predicted performance. Furthermore, possibly because in this study we recruited participants with aphasia who had extremely poor naming performance, we also observed a group difference: participants with aphasia had lower accuracy and longer response times than the two control groups. This evidence points to a possible link between naming performance and categorization. As in Study 1, this link might arise from the fact that task instructions are presented verbally; thus, linguistic impairments might affect task performance simply because they make it more challenging to process the instructions. Another explanation, also offered by L&M, is that LD categorization is correlated with naming impairments because both tasks may be affected by damage to cognitive control mechanisms, which lay in close proximity to language areas, especially in the LIFG (Thompson-Schill et al. 1997; Kan and Thompson-Schill 2004; Fedorenko et al. 2012). In line with this conjecture, Hu et al. (2021) observed strong neural responses (in fMRI) in the domain-general MD network during an object naming task. Thus, the correlation between anomia severity and object categorization performance does not offer evidence of a language-specific impairment and might reflect an executive impairment instead.

The results of Studies 1 and 2 did not allow us to resolve the question of whether language plays a key role in LD categorization. Study 1 failed to replicate the selective LD categorization impairments as reported in L&M. Study 2 did show a selective decrease in accuracy (and increase in RTs) for LD categories in participants with low naming scores, as predicted by the LD-specific language recruitment hypothesis. However, this piece of evidence is undermined by the dissociation observed in participants A4 and A5 and the possibility that the performance deficits in individuals with severe anomia could be caused by damage to the domain-general executive brain regions that are adjacent to the language system in the left frontal lobe.

To definitively establish whether LD categorization recruits the language system, we next turned to fMRI.

## fMRI experiment

To further test the relationship between language and categorization, we conducted an fMRI experiment. Neurotypical participants performed the same LD/HD categorization task as participants in Study 2. In addition, they completed two functional “localizer” tasks (Saxe et al. 2006; Fedorenko et al. 2010) that were used to identify the networks of interest: the language network and the multiple demand network. The use of standard, extensively validated language network and multiple demand networks localizers allows us to identify and characterize these networks consistently across studies (Saxe et al. 2006; Fedorenko 2021).

The language localizer was designed to identify brain regions that respond more strongly to meaningful and structured language than a perceptually similar control condition (for example, sentences versus meaningless sequences of letters (“nonwords”); Fedorenko et al. 2010). A large number of studies have shown that sentences>nonwords and similar contrasts pick out a set of brain regions that are strongly and selectively recruited for language processing, including spoken, written, and signed language comprehension, spoken and written language production, and inner speech (Amit et al. 2017; Braga et al. 2020; Fedorenko et al. 2010, 2011; Giglio et al. 2022; Hu et al. 2021; Menenti et al. 2011; Scott et al. 2017; Silbert et al. 2014). These regions (henceforth, the language network) also respond to linguistic units at different levels of the processing hierarchy, including both phrases and single words (albeit no single region or voxel is sensitive just to word-level or sentence-level meaning; Blank et al. 2016; Fedorenko et al. 2020). Therefore, if a task requires activating verbal labels, we expect to observe activity in the regions identified with the language localizer.

The multiple demand localizer identifies a set of brain regions that respond to a wide range of cognitively demanding tasks. Specifically, these regions are sensitive to general cognitive effort, exhibiting higher activity when the task is more difficult (Assem et al. 2020b; Duncan 2010; Fedorenko et al. 2013; Hugdahl et al. 2015). The hard>easy response signature in the multiple demand network holds across many diverse tasks, including spatial WM, logic, math, relational reasoning, and cognitive control (Fedorenko et al. 2013; Coetzee and Monti 2018; Shashidhara et al. 2019; Assem et al. 2020b). Thus, if LD categorization is more cognitively challenging, we expect it to elicit higher activity in the multiple demand network.

Examining activation patterns in both the language and the multiple demand networks allows us to examine the relative contributions of linguistic and cognitive control resources to LD and HD categorization. As discussed before, brain damage leading to aphasia is often comorbid with multiple demand network damage: the language-selective regions and these domain-general regions in left inferior frontal cortex lie in close proximity to each other (Fedorenko et al. 2012; Blank et al. 2014; Fedorenko and Blank 2020), with precise locations varying substantially across individuals. Thus, impaired categorization performance of participants with aphasia in Studies 1 and 2 could have potentially arisen from damage to either or both networks. Study 3 allows us to disambiguate between these possibilities. If, as suggested by L&M, LD categorization indeed relies on language more than HD categorization, we expect to see more activity within the language system during LD trials compared with HD trials. Further, if LD categorization is a more cognitively demanding task, we expect to see higher responses within the multiple demand network during LD trials compared with HD trials (in accordance with the fact that multiple demand regions are sensitive to effort across diverse tasks; Duncan and Owen 2000; Fedorenko et al. 2013; Hugdahl et al. 2015). Finally, if a brain network does not respond to either LD or HD categorization, we can conclude that this network is not recruited for this task.

### Method

#### Participants

Fourteen neurotypical participants (7 F, age *M* = 22.31, *SD* = 3.51) were recruited from MIT and the surrounding community and paid $60 for their participation. All were native speakers of English. One participant was left-handed (see Willems et al. 2014, for motivation to include left-handers in cognitive neuroscience research) but showed typical left-lateralized language activation as determined by the language localizer task (described below). All participants gave informed consent in accordance with the requirements of MIT’s Committee On the Use of Humans as Experimental Subjects.

#### Design, materials, and procedure

Each participant completed a language localizer task aimed at identifying language-responsive brain regions (Fedorenko et al. 2010), a spatial WM task aimed at identifying the multiple demand network (Fedorenko et al. 2013) and the critical categorization task. Some participants completed one or more additional tasks for unrelated studies. The entire scanning session lasted two hours.

##### Language network localizer

Participants read sentences (e.g. NOBODY COULD HAVE PREDICTED THE EARTHQUAKE IN THIS PART OF THE COUNTRY) and lists of unconnected, pronounceable nonwords (e.g. U BIZBY ACWORRILY MIDARAL MAPE LAS POME U TRINT WEPS WIBRON PUZ) in a blocked design. Each stimulus consisted of twelve words/nonwords. The sentences > nonword-lists contrast has been previously shown to reliably activate high-level language processing regions and to be robust to changes in the materials, task, and modality of presentation (Fedorenko et al. 2010; Mahowald and Fedorenko 2016; Scott et al. 2017). For details of how the language materials were constructed, see Fedorenko et al. (2010). The materials are available at http://evlab.mit.edu/funcloc. Stimuli were presented in the center of the screen, one word/nonword at a time, at the rate of 450 ms per word/nonword. Each stimulus was preceded by a 100 ms blank screen and followed by a 400-ms screen showing a picture of a finger pressing a button, and a blank screen for another 100 ms, for a total trial duration of 6 s. Participants were asked to press a button whenever they saw the picture of a finger pressing a button. This task was included to help participants stay alert and awake. Condition order was counterbalanced across runs. Experimental blocks lasted 18 s (with theree trials per block), and fixation blocks lasted 14 s. Each run (consisting of 5 fixation blocks and 16 experimental blocks) lasted 358 s. Each participant completed two runs.

##### Multiple demand network localizer

Participants had to keep track of four (easy condition) or eight (hard condition) sequentially presented locations in a 3 × 4 grid (Fedorenko et al. 2013). The hard > easy contrast has been previously shown to robustly activate multiple demand regions (Fedorenko et al. 2013; Blank et al. 2014; Mineroff et al. 2018; Assem et al. 2020a). Stimuli in both conditions were presented in the center of the screen across four steps. Each of these steps lasted for 1 s and presented one location on the grid in the easy condition, and two locations in the hard condition. Each stimulus was followed by a choice-selection step, which showed two grids side by side. One grid contained the locations shown on the previous four steps, whereas the other contained an incorrect set of locations. Participants were asked to press one of two buttons to choose the grid that showed the correct locations. Condition order was counterbalanced across runs and participants. Experimental blocks lasted 32 s (with 4 trials per block), and fixation blocks lasted 16 s. Each run lasted 448 s, consisting of 12 experimental blocks (6 per condition) and 4 fixation blocks. Twelve participants completed two runs and two participants completed one run.

##### Critical categorization task

The categorization materials were the same as those used in Study 2 (see Fig. 1, bottom). The timing differed in the following way. In order to make blocks uniform in duration, each category block started with a category label presented for 2 s, and then the 12 images were presented sequentially at the fixed speed of 2 s per image. As in Study 2, any given category block contained between four and six target images. Participants were asked to press a button if the picture belonged to the target category and not to press anything if it did not. As before, the category label was displayed at the top of the screen for the duration of the trial to minimize memory demands. Category blocks lasted 26 s (2 s category label presentation +2 s × 12 images), and fixation blocks lasted 14 s. Each run, consisting of 12 category blocks (6 LD and 6 HD) and 4 fixation blocks, lasted 368 s. Each participant completed three runs. Across the three runs, any given participant saw a random subset of the 32 categories, with some categories repeating (but never repeating within a run; see Appendix S1, Table 1 for details). Condition order was counterbalanced across runs and participants.

#### fMRI data acquisition

Structural and functional data were collected on the whole-body, 3 Tesla, Siemens Trio scanner with a 32-channel head coil, at the Athinoula A. Martinos Imaging Center at the McGovern Institute for Brain Research at MIT. T1-weighted structural images were collected in 176 sagittal slices with 1-mm isotropic voxels (TR = 2,530 ms, TE = 3.48 ms). Functional, blood oxygenation level dependent (BOLD), data were acquired using an EPI sequence (with a 90 ° flip angle and using GRAPPA with an acceleration factor of 2), with the following acquisition parameters: 31 4-mm thick near-axial slices acquired in the interleaved order (with 10% distance factor), 2.1 mm × 2.1 mm in-plane resolution, FoV in the phase encoding (A> > P) direction 200 mm and matrix size 96 mm × 96 mm, TR = 2000 ms and TE = 30 ms. The first 10s of each run were excluded to allow for steady state magnetization.

#### fMRI data preprocessing

fMRI data were analyzed using SPM12 (release 7487), CONN EvLab module (release 19b), and other custom MATLAB scripts. Each participant’s functional and structural data were converted from DICOM to NIFTI format. All functional scans were coregistered and resampled using B-spline interpolation to the first scan of the first session (Friston Karl et al. 1995). Potential outlier scans were identified from the resulting subject-motion estimates, as well as from BOLD signal indicators, using default thresholds in CONN preprocessing pipeline (5 standard deviations above the mean in global BOLD signal change, or framewise displacement values above 0.9 mm; Nieto 2020), and used as regressors of no interest in first-level analyses (see below). Functional and structural data were independently normalized into a common space [the Montreal Neurological Institute (MNI) template; IXI549Space[ using SPM12 unified segmentation and normalization procedure (Ashburner and Friston 2005) with a reference functional image computed as the mean functional data after realignment across all timepoints omitting outlier scans. The output data were resampled to a common bounding box between MNI-space coordinates (−90, −126, −72) and (90, 90, 108), using 2-mm isotropic voxels and fourth-order spline interpolation for the functional data, and 1-mm isotropic voxels and trilinear interpolation for the structural data. Last, the functional data were smoothed spatially using spatial convolution with a 4-mm FWHM Gaussian kernel.

#### First-level analysis

Responses in individual voxels were estimated using a General Linear Model (GLM) in which each experimental condition was modeled with a boxcar function convolved with the canonical hemodynamic response function (HRF) (fixation was modeled implicitly, such that all timepoints that did not correspond to one of the conditions were assumed to correspond to a fixation period). Temporal autocorrelations in the BOLD signal timeseries were accounted for by a combination of high-pass filtering with a 128-s cutoff and whitening using an AR(0.2) model (first-order autoregressive model linearized around the coefficient *a* = 0.2) to approximate the observed covariance of the functional data in the context of Restricted Maximum Likelihood estimation (ReML). In addition to experimental condition effects, the GLM design included first-order temporal derivatives for each condition (included to model variability in the HRF delays), as well as nuisance regressors to control for the effect of slow linear drifts, subject-motion parameters, and potential outlier scans on the BOLD signal.

#### Defining individual functional regions of interest

Responses to the critical categorization experiment were extracted from regions of interest that were defined functionally in each individual participant (Saxe et al. 2006; Nieto-Castañón and Fedorenko 2012). Three sets of functional regions of interest (fROIs) were defined—one for the language network, one for the multiple demand network, and one for the putative LD > HD categorization regions. To do so, we used the Group-constrained Subject-Specific (GSS) approach (Fedorenko et al. 2010; Julian et al. 2012). In particular, fROIs were constrained to fall within a set of “parcels,” which marked the expected gross locations of activations for the relevant contrast. For the language network, the parcels were generated based on a group-level representation of language localizer data from 220 participants. For the multiple demand network, the parcels were generated based on a group-level representation of spatial WM task data from 197 participants. For the putative LD categorization regions, we generated the parcels based on the data collected in this study. The parcels are available on OSF (https://osf.io/guwh8/).

To create each set of parcels, individual activation maps for the relevant localizer contrast were binarized (by turning all voxels significant at the *P* < 0.001 whole-brain threshold (uncorrected) into 1 s, and the rest into 0 s) and overlaid in the MNI space to create a probabilistic overlap map. The map was then smoothed (FWHM = 6 mm), and voxels with fewer than 10% of participants overlapping were excluded. The resulting map was divided into regions using a watershed algorithm. Finally, we excluded parcels that did not show significant effects for the relevant localizer contrast in a left-out run or did not contain supra-threshold voxels in at least 60% of the participants (for language and multiple demand networks) or in at least 50% of the participants (for putative LD categorization regions). For the multiple demand network, we also (i) excluded parcels in the visual cortex (the hard condition includes more visual information than the easy condition and thus yields more activation in the visual cortex), and (ii) divided a parcel that encompassed parts of both the precentral gyrus and the opercular portion of the inferior frontal gyrus according to the macroanatomical boundary.

For each participant, each set of masks was intersected with the participant’s activation map for the relevant contrast (sentences>nonwords for the language network, hard>easy spatial WM for the multiple demand network, and LD > HD for putative LD categorization regions). Within each mask, the voxels were sorted based on their *t-*values for the relevant contrast, and the top 10% of voxels were selected as that participant’s fROI. This top *n*% approach ensures that the fROIs can be defined in every participant, thus enabling us to generalize the results to the entire population (Nieto-Castañón and Fedorenko 2012).

#### Examining the functional response profiles of fROIs

After defining fROIs in individual participants, we evaluated their responses to the conditions of interest by averaging the responses across voxels to get a single value per condition per fROI. This fROI-level estimate of the BOLD response magnitude is our main effect of interest in this study (and the response magnitude averaged across participants constitutes a measure of the effect size).

The responses to the localizer conditions (sentences and nonwords for language fROIs, hard and easy WM conditions for multiple demand fROIs, and LD and HD categorization for categorization fROIs) were estimated using an across-runs cross-validation procedure, where one run was used to define the fROI and the other to estimate the response magnitudes, then the procedure was repeated switching the runs used for fROI definition versus response estimation, and finally the estimates were averaged to derive a single value per condition per fROI per participant. This cross-validation procedure allows one to use all of the data for defining the fROIs as well as for estimating their responses (see Nieto-Castañón and Fedorenko 2012, for discussion), while ensuring the independence of the data used for fROI definition and response estimation (Kriegeskorte et al. 2009). Two participants completed only one run of the multiple demand localizer task; therefore, we did not estimate the strength of their responses to the hard and easy multiple demand localizer conditions but ensured that the whole-brain activation maps for the hard>easy contrast showed the expected topography.

#### Statistical analyses

Similar to Studies 1 and 2, we analyzed our data using mixed effect regression models (Baayen et al. 2008). For accuracy, we use logistic regression (Jaeger 2008). For RT and fROIs response magnitudes, we use linear regression. In all models, condition was a fixed effect and participant was a random intercept. The model for the multiple demand network included hemisphere as an additional fixed effect. For language and multiple demand network analyses, we also included fROI as a random intercept and then ran follow-up analyses on individual fROIs using false discovery rate (FDR) correction (Benjamini and Hochberg 1995) for the number of fROIs in each network. Behavioral analyses used sum coding for condition (LD vs. HD in the categorization task and Hard vs. Easy in the multiple demand localizer task). Neuroimaging analyses used custom contrasts (see Appendix 3 for detailed contrast specification). The mixed effect analyses were run using the *lmer* function from the *lme4* R package (Bates et al. 2015); statistical significance of the effects was evaluated using the *lmerTest* package (Kuznetsova et al. 2017). The hypotheses-specific contrasts were defined using the *hypr* package (Rabe et al. 2020).

In sum, if linguistic resources are engaged during categorization, we would expect an overall high response of the language network to categorization conditions. Further, if, as L&M have argued, LD categorization taxes linguistic resources to a greater extent, we would expect to see stronger response of this network to the LD compared with the HD condition. Lastly, if LD categorization is generally more taxing, we would expect to see greater responses to the LD condition in the domain-general multiple demand regions that are sensitive to effort across diverse tasks (Duncan 2010; Duncan 2013; Fedorenko et al. 2013; Hugdahl et al. 2015).

## Results

### Behavioral data

#### Multiple demand network localizer

Due to a technical error, behavioral data for one participant got overwritten. For the remaining thirteen participants, performance on the spatial WM task was as expected: participants were more accurate and faster in the easy condition (accuracy *M* = 93.91%, *SD* = 3.00%; reaction time (RT) = 1.18 s, *SD* = 0.16 s) than the hard condition (accuracy *M* = 79.65%, *SD* = 12.03%; RT *M* = 1.52 s, *SD* = 0.25 s). Mixed effect models with condition as a fixed effect and participant as a random intercept showed that both accuracy and RT effects were significant (accuracy: β = −1.41, SE = 0.202, *P* < 0.001; RT: β = 0.33, *SE* = 0.027, *P* < 0.001).

##### Critical categorization task

The accuracies for the two categorization conditions did not significantly differ (LD *M* = 95.73%, *SD* = 4.20%; HD *M* = 95.44%, *SD* = 4.11%; LD > HD β = 0.14, *SE* = 0.20, *P* = 0.454). Similarly, there was no significant difference between response times in the LD condition (RT = 0.81 s, *SD* = 0.1 s) and the HD condition (RT = 0.84 s, *SD* = 0.1 s; LD > HD β = −0.03, *SE* = 0.02, *P* = 0.156).

#### Functional response profile of the language network

**Fig. 4:**
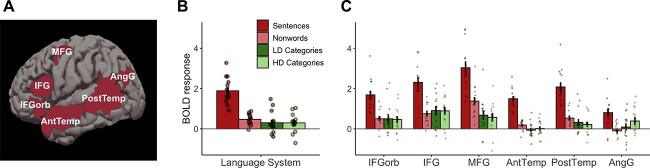
Categorization responses within the language brain network. (A) Parcels used to define fROIs in individual participants. (B) Average responses within the language network to four conditions of interest (sentence reading and nonword reading vs. LD and HD categorization). (C) fROI responses to the four conditions of interest.

There was no significant difference between language network responses to LD and HD categorization (β = −0.02, *SE* = 0.10, *P* = 0.848). Overall, responses to the categorization task were barely above 0 (β = 0.42, *SE* = 0.19, *P* = 0.054; see Fig. 4), not significantly different from responses to nonword reading, the control condition in the language localizer task (β = 0.13, *SE* = 0.09, *P* = 0.144), and significantly weaker than responses to sentences (β = −1.49, *SE* = 0.09, *P* < 0.001).

Follow-up analyses in individual language fROIs (Appendix 2, Table 1) showed that responses to categorization were significantly above 0 in frontal fROIs (MFG, IFG, and IFGorb). However, none of the responses were significantly higher than responses during the control task, nonword reading, indicating that these responses are not language-specific. Thus, our results suggest that the language network does not support either LD or HD categorization in neurotypical participants.

#### Functional response profile of the multiple demand network

**Fig. 5:**
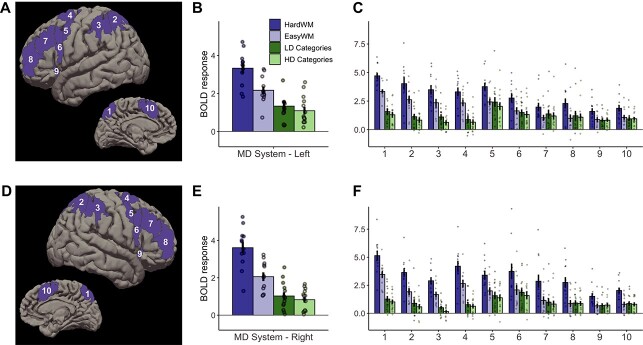
Categorization responses within the multiple demand brain network. (A) Left hemisphere parcels used to define fROIs in individual participants. (B) Average responses within the left hemisphere fROIs to four conditions of interest (hard and easy WM tasks vs. LD and HD categorization). (C) Left hemisphere fROI responses to the four conditions of interest. (D–F) Parcels, average responses, and fROI-level responses in the right hemisphere.

Multiple demand network response to LD categorization was higher than to HD categorization (β = 0.19, *SE* = 0.09, *P* = 0.025), indicating that, as predicted, LD categorization is more effortful. In general, multiple demand network responses to categorization were significantly above 0 (β = 1.07, *SE* = 0.21, *P* < 0.001; see Fig. 5) and stronger than responses to control conditions from the language localizer task (categorization > sentences: β = 0.73, *SE* = 0.08, *P* < 0.001; categorization > nonwords: β = 0.41, *SE* = 0.08, *P* < 0.001). However, they were weaker than responses to the spatial WM task (β = −1.43, *SE* = 0.07, *P* < 0.001), indicating that the WM task was more effortful. Responses to the categorization task were stronger in the left hemisphere (β = 0.24, *SE* = 0.09, *P* = 0.005). We also observed an interaction between the WM > categorization contrast and hemisphere (β = 0.29, *SE* = 0.13, *P* = 0.024), showing that the WM task engages the right hemisphere to a greater extent. There was also an interaction between the Hard>Easy WM task and hemisphere, such that the effect was greater in right hemisphere (β = 0.38, *SE* = 0.19, *P* = 0.040).

Follow-up analyses on individual fROIs (Appendix 2, Table 2) showed that responses to categorization were significantly above 0 in all fROIs. However, they were weaker than the overall responses to the WM task in almost all fROIs (except left middle frontal fROI). This result highlights the domain-general nature of these responses. Further, none of the fROIs had significantly different responses to LD and HD categories, despite the presence of this effect in the network-level analysis.

#### Whole-brain analyses

**Fig. 6:**
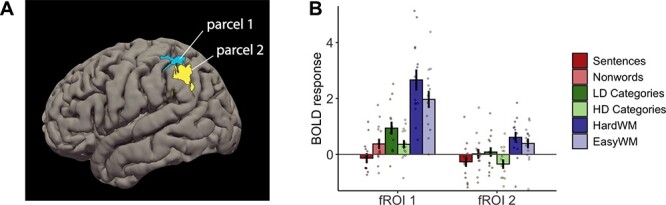
Results of the whole-brain analyses. (A) Parcels defined with the LD > HD categorization contrast. (B) Responses to conditions of interest within the two fROIs (defined as the top 10% of voxels within each parcel, sorted by the magnitude of the LD > HD response). WM, working memory task.

We also conducted a whole-brain analysis to identify fROIs that might respond more strongly to LD or HD categorization but lie outside the language and multiple demand fROIs described above. The GSS analysis (see Methods for details) revealed that no regions exhibited consistent HD > LD responses across participants; however, the LD > HD contrast revealed two parcels, both located in left parietal lobe (Fig. 6). Further analysis of fROIs defined within these parcels showed that the LD > HD response only reached significance in fROI 2 (β = 0.43, *SE* = 0.17, *P* = 0.013), but not in fROI 1 (β = 0.58, *SE* = 0.30, *P* = 0.060). The overall categorization response was significantly above 0 in fROI 1 (β = 0.65, *SE* = 0.19, *P* = 0.001) but not fROI 2 (β = −0.13, *SE* = 0.15, *P* = 0.389).

Importantly, both fROIs responded to the WM task more strongly than to the categorization task (fROI 1: β = 1.66, *SE* = 0.21, *P* < 0.001; fROI 2: β = 0.64, *SE* = 0.12, *P* < 0.001), indicating that these regions likely respond to general cognitive effort rather than to LD categorization (or feature selection) specifically, and thus likely belong to the MD network. Neither of the two fROIs exhibited a sentences>nonwords effect; in fact, both showed a trend in the opposite direction (fROI 1: β = −0.51, *SE* = 0.30, *P* = 0.094; fROI 2: β = −0.28, *SE* = 0.17, *P* = 0.098), which shows that these regions do not respond to linguistic input.

The whole-brain analysis provides additional evidence against the LD-specific language recruitment hypothesis and shows that differences in LD versus HD categorization, if present, are likely caused by domain-general mechanisms.

### Interim discussion

In the fMRI Experiment, we examined neural responses to LD and HD categorization. Our main goal was to evaluate the hypothesis that LD categorization relies more heavily on linguistic resources compared with HD categorization. For this purpose, we identified the language network individually in 14 healthy adults and examined its responses during LD and HD categorization. The language network exhibited low responses to both categorization tasks, which did not differ from activations elicited by reading of nonword sequences (a low-level control condition). There was no difference between responses to LD and HD categories, contrary to the prediction that the language network would be selectively or preferentially engaged during LD categorization. Thus, we conclude that (i) the neuroimaging results disconfirm the LD-specific language recruitment hypothesis and (ii) the language network is not at all engaged in object categorization, highlighting a dissociation between linguistic processing and non-linguistic semantic cognition.

Unlike the language network, the domain-general multiple demand network (also defined individually in each participant) was engaged during categorization, indicating that this task is cognitively challenging. This network responded more strongly to LD than HD categorization, but this effect was small. The whole-brain analyses specifically aimed at identifying regions with stronger responses to LD than HD categorization confirmed that the two identified fROIs, responded more strongly to a WM task than to a categorization task, and the LD > HD effect was small and/or not statistically significant. We conclude that categorization, and LD categorization in particular, relies on domain-general multiple demand regions and not on language-specific regions. Future work should examine whether the small difference between LD and HD categories is driven by a small subset of categories or whether it indeed reflects greater domain-general cognitive demands associated with all LD categorization.

Neuroimaging of healthy individuals provides a powerful complement to patient studies. Given the strong and selective engagement of the language network during all behaviors requiring access to linguistic representations (Fedorenko et al. 2010; Fedorenko et al. 2011; Menenti et al. 2011; Scott et al. 2017; Giglio et al. 2022; Hu et al. 2021, among others), the lack of activity in the language regions during categorization strongly suggests that they do not contribute to categorization (Mather et al. 2013). The response to categorization within the multiple demand network, on the other hand, indicates its involvement in categorization, even though we note that fMRI evidence described here is correlational, not causal, and should be complemented with patient studies or brain stimulation studies that specifically target this hypothesis (that interfering with the activity in the multiple demand network or damage to this network should lead to impairments in categorization tasks). Neuroimaging evidence is particularly helpful when patient studies do not produce conclusive results, as in our case.

Whereas some previous work suggested that a region within left angular gyrus is involved in inhibiting irrelevant semantic information (Lewis et al. 2019), as may be required for LD categorization, the results of our study suggest that activation of the language-responsive portion of the left angular gyrus was comparable during LD and HD categorization. If anything, this language fROI showed numerically higher activation during HD categorization, suggesting that it may be recruited for recognizing and thinking about established sets more than for constructing novel sets that may require inhibition of object-irrelevant characteristics. We also did not find significant differences in the engagement of the language fROIs in the left inferior frontal cortex during LD and HD categorization. These results are in contrast to findings from Lupyan et al. (2012), which suggested that tDCS to the left inferior frontal cortex disrupted performance on LD but not HD categorization. The latter result might be explained by the fact that left inferior frontal cortex contains not only language-responsive areas, but also multiple demand areas (Fedorenko et al. 2012; Fedorenko and Blank 2020), and interfering with the latter areas’ activity may have a disproportionately higher effect on LD categorization.

The response to categorization within the multiple demand network was stronger in the left hemisphere, consistent with the view that label-based categorization recruits the left hemisphere more strongly (Gilbert et al. 2006; Franklin et al. 2008). This makes the categorization task similar to logic and math, which also evoke left-lateralized responses within the multiple demand network (Monti et al. 2009; Pinel and Dehaene 2009; Monti et al. 2012; Amalric and Dehaene 2016). Importantly, our result demonstrates that, just because the function is left-lateralized, it is not necessarily related to language, at least not in fully formed brains (contra, e.g. Gilbert et al. 2006; see also Holmes and Wolff 2012).

All in all, results from the fMRI Experiment disconfirm the hypothesis that LD categorization relies on linguistic resources. Instead, they show that categorization recruits the multiple demand brain regions and that LD categorization is, on average, slightly more effortful that HD categorization.

## Alternative account: semantic versus perceptual categories

Throughout this paper, we have adopted the LD/HD distinction proposed by L&M and tested their hypothesis using the same categories as those in their study. However, the LD/HD distinction might not be the only relevant distinction for testing the role of language in object categorization (see Section 6.2 for potential issues with this classification scheme). Therefore, we additionally tested an alternative hypothesis: that the language network would be selectively recruited for processing semantic categories (e.g. DANGEROUS ANIMALS) but not perceptual categories (e.g. THINGS THAT ARE BLUE). This classification does not fully align with the HD/LD distinction and instead reflects the view that language and semantic, or conceptual, processing are tightly linked (see, e.g. Binder et al. 2009; Binder and Desai 2011; cf. Patterson et al. 2007; Ivanova et al. 2021).

### Method

We re-analyzed the data from the two aphasia studies and the fMRI experiment by re-coding the categories as either semantic or perceptual. The criterion we used was the following. For perceptual categorization, one does not need to know the identity of the object because the information required for categorization (e.g. color, length) is directly extractable from the image. For semantic categorization (e.g. danger level or typical location), however, the identity of the object is important. The result of this re-coding is reported in Appendix 1. The rest of the analyses were the same as those described for LD/HD category types.

### Results and discussion

The results are shown in Appendices S2 and S3. In both aphasia studies, category type had no effect on accuracy, nor did it interact with participant group or BNT. However, semantic categorization overall elicited longer response times compared with perceptual categorization. This main effect of category type on response times interacted with participant group for both studies, but the interaction went in opposite directions across studies: in Study 1, individuals with low BNT showed an increased difference in response time between semantic and perceptual categories, whereas in Study 2, this gap was reduced. The results of the aphasia studies are therefore inconclusive but do not provide support for a consistent relationship between naming ability and categorization.

The neuroimaging results, however, are clear. The language network is not significantly recruited for either semantic or perceptual categories, reinforcing our conclusion that the cognitive mechanisms responsible for core language processing are not engaged in object categorization.

Given that the semantic nature of the category has an effect on response times during categorization tasks, future works should aim to disentangle category dimensionality and semantic content when designing the stimuli.

## General discussion

We reported three studies that evaluated the hypothesis that linguistic resources are essential for performing feature-based, or LD, categorization—what we refer to as the “LD-specific language recruitment hypothesis” (Lupyan 2009; Lupyan et al. 2012; Lupyan and Mirman 2013; Langland et al. 2021). In Study 1, we aimed to replicate the results of Lupyan and Mirman (2013), who showed a selective impairment in LD categorization in individuals with aphasia. Our results failed to replicate this critical finding, although they did show that naming ability, as measured by BNT scores, was a significant predictor of overall categorization performance.

In Study 2, we modified the design to reduce general task complexity and examined the specific contribution of naming ability to categorization by recruiting a group of participants with very low naming scores. We found that, in accordance with the LD-specific language recruitment hypothesis, individuals with aphasia were more impaired on LD compared with HD categorization. However, a case-by-case analysis revealed that two individuals with a severe naming impairment (with scores of 1 and 4 out of 60 on the BNT) performed within the neurotypical range on both HD and LD categorization. Evidence from patients with brain lesions remains an important way to establish whether specific cognitive capacities support performance on particular tasks (Rorden and Karnath 2004), and dissociations are more important than associations in this kind of evidence (Caramazza and Coltheart 2006). Patient studies have previously demonstrated that many high-order cognitive functions are not affected by even severe linguistic deficits (e.g. Apperly et al. 2006; Bek et al. 2013; Chen et al. 2020; Varley et al. 2001, 2005; Varley and Siegal 2000; Willems et al. 2011; Ivanova et al. 2021). Based on Study 2, we therefore concluded that lexical retrieval is *not necessary* for successful categorization, including categorization based on single features.

In Study 3, we used a complementary approach and examined the engagement of the language network and a domain-general multiple demand network in HD and LD categorization using fMRI in neurotypical adults. The language network was not engaged during either LD or HD categorization: its responses did not significantly differ from responses during the control, nonword reading, task. This observation goes against the hypothesis that categorization (either LD or HD) relies on linguistic resources. In contrast, the multiple demand network was recruited during the categorization task, consistent with prior evidence of its involvement in diverse cognitively challenging tasks (Duncan 2010; Duncan 2013; Fedorenko et al. 2013; Assem et al. 2020b). It also responded more strongly during LD than HD categorization. Given extensive evidence that the multiple demand network responds more strongly when the task is harder (e.g. Fedorenko et al. 2011; Fedorenko et al. 2013; Hugdahl et al. 2015; Shashidhara et al. 2019), the increased response during LD categorization is consistent with the hypothesis that LD categorization is more cognitively challenging. However, this effect was small and did not come out as statistically significant in any of the individual multiple demand regions in follow-up analyses. In sum, we find little evidence in favor of the LD-specific language recruitment hypothesis.

### The cognitive control account of categorization performance

The failure to replicate the results from L&M in Study 1 and an only partial replication in Study 2 have several possible explanations. The first explanation is that the effect described by L&M is real, but we could not detect it due to low power (e.g. small sample size). This explanation is unlikely because of our neuroimaging results: if language was indeed required for LD categorization, the language network would be active during the LD categorization condition. The second explanation is that the result that was reported by L&M is a false positive. The third explanation is that the effect holds in a subset of individuals with aphasia, due to comorbid cognitive control impairments. We cannot definitively rule out either the second or the third explanation, although our neuroimaging results provide some support for the latter: the multiple demand network, implicated in cognitively demanding tasks, was somewhat more active during LD than during HD categorization.

The hypothesis that domain-general cognitive control deficits underlie impaired categorization can also explain the link between categorization and naming, which we observed in both Studies 1 and 2, and which was also reported by L&M. Confrontation naming is a complex, multi-component behavior that involves not only linguistic, but also visual, motor-articulatory, and critically, executive resources. Indeed, a recent fMRI study (Hu et al. 2021) reports strong responses within the multiple demand network to an object naming condition. Furthermore, unlike syntactic comprehension, both naming ability and fluid intelligence (a trait linked to the multiple demand network; Gläscher et al. 2010; Woolgar et al. 2010; Woolgar et al. 2018) decline with age, and this decline is linked to decreased activity in the multiple demand brain regions during both of these tasks (Samu et al. 2017). Thus, although both our work and L&M show a relationship between naming and categorization, the underlying cognitive mechanism of this relationship is likely related to cognitive control, not language.

Yet another possibility is that both naming and categorization performance rely not only on domain-general, but also on semantic control resources. Semantic control is a cognitive construct posited by several groups that investigate controlled retrieval of conceptual information (e.g. Thompson-Schill et al. 1997; Badre and Wagner 2002; Jefferies 2013; Lambon Ralph et al. 2017). Although the location of the putative regions responsible for semantic control (or, more neutrally, semantic demand) resembles that of the language regions, precise localization approaches in individual brains indicate that language, multiple demand, and semantic demand regions are spatially distinct (Ivanova et al. in prep). If semantic demand regions support deliberate, controlled semantic tasks, damage to these regions might explain both categorization and naming difficulties in individuals with anomia. However, that would not constitute evidence in favor of the LD-specific language recruitment hypothesis: semantic demand regions get recruited both for verbal and nonverbal inputs (Ivanova et al. in prep) and are therefore not language-specific.

Future patient studies should explicitly test the cognitive control accounts of LD-selective categorization impairments. One way to do so is to use lesion mapping along with probabilistic maps of functional networks of interest (see, e.g. Woolgar et al. 2018): this method allows explicitly determining which network (language, multiple demand, or semantic control) underlies observed behavior patterns. Another way is to measure domain-general and semantic cognitive control in individuals with brain damage and use them as predictors when evaluating the relationship between naming performance and categorization. Yet another approach would be to explore these relationships in neurotypical participants by examining the correlational structure of these abilities across individuals. Such studies could provide additional evidence in favor or against the cognitive control accounts of categorization impairments, complementing our neuroimaging results and reconciling conflicting findings from individuals with aphasia.

### The relevance of LD versus HD distinction

Why did we find no, few, or inconsistent differences in performance and neural responses between LD and HD categories? A possible explanation is that “LD” and “HD” category types are not “natural kinds.” In the interest of replicability, we here chose to keep the categories used by L&M for most analyses, but future research will possibly refine or even abandon this distinction. As discussed in the introduction, different researchers have emphasized different distinctions among categories, such as natural/ad hoc, taxonomic/thematic, dense/sparse, concrete/abstract, etc. Many of these distinctions are not isomorphic with the LD/HD distinction. In particular, HD categories encompass both taxonomic (e.g. “animals”) and thematic (e.g. “non-food things found in the kitchen”) categories. Multiple studies show that the processing of taxonomic and thematic relations relies on distinct cognitive and neural mechanisms (e.g. Kalénine et al. 2009; Sass et al. 2009; Schwartz et al. 2011; Lewis et al. 2015; Xu et al. 2018); collapsing them into a single “HD” category type leads to substantial within-HD heterogeneity and may therefore obscure potential HD/LD differences.

In addition, there is currently no principled way of labeling categories as LD versus HD. Different researchers might disagree on whether items in a given category have few or many features in common: for instance, Lupyan and Mirman (2013) classify “things that fly” as an HD category, even though the majority of members in this category can be identified using an LD label “have wings”; under other accounts (e.g. Langland et al. 2021), “flying” might be a feature in and of itself, uniting objects that are otherwise highly diverse. The lack of clarity on what exactly constitutes an HD category makes it hard to generalize the results beyond the specific categories used in the study.

Furthermore, not all LD categories as defined by Lupyan and Mirman (2013) necessarily involve conceptual processing. For instance, many are based on color: e.g. “THINGS THAT ARE YELLOW”. Although color is often encoded as part of the conceptual representation of an object, this conceptual representation was not required for the task in question: participants were simply asked to indicate whether the object they were viewing was yellow, and decisions could be made on the basis of surface perceptual features alone. Thus, even if “true” (semantic) LD categories are indeed harder to process than HD categories, inclusion of perception-based color categories could have prevented us from reliably observing this difference.

Our results are somewhat inconsistent with recent work by Langland et al. (2021), who observe that individuals with aphasia were slower and less accurate (compared with healthy adults) when processing abstract categories compared with concrete categories. The authors argue that the abstract/concrete distinction is similar to the LD/HD distinction because members of abstract categories share fewer common features. However, another important difference is the *kind* of features used for categorization. For instance, their example of an abstract category “predict” (which includes a weatherperson and a fortune-teller) relies on an unobservable functional similarity rather than on an observable visual similarity. Unobserved features play an important role in the use of verbal category labels (Gelman and Roberts 2017), so it is possible that language mediates categorization based on *latent* features rather than LD categorization per se. In short, the LD/HD and the abstract/concrete distinction do not cleanly map onto each other, which makes it difficult to compare the results of our studies to those by Langland-Hassan et al. More generally, the typology of category types remains vague and inconsistent, and more careful work should be done to establish meaningful category distinctions and thus facilitate comparisons across studies.

### Possible paradigm-specific effects of verbal labels

Even if we were able to successfully replicate L&M’s findings, our conclusions about the language–categorization link would be complicated by the fact that the paradigm introduced by L&M is not language-free. In order to successfully sort objects into categories, participants need to read (or hear) and encode the category label, presented verbally. The importance of language during the instruction encoding stage might account for the relationship between categorization performance and naming ability; it might even explain the (putative) LD-specific categorization impairments, given that category labels for LD categories are often longer. In Studies 2 and 3, we simplified the visual processing demands, and separated the category-label instruction from the task, which allowed us to measure the behavioral and neural responses to categorization more clearly. Another solution to this issue would be to modify the paradigm to remove verbal labels altogether, e.g. by providing several category exemplars instead.

In addition, linguistic labels might contribute to the task via verbal rehearsal: participants might employ a phonological loop to maintain an active representation of the labels in WM. Such assistive role of language labels has been observed in conditions of high cognitive demand (e.g. during mathematical calculation; Benn et al. 2012; Klessinger et al. 2012). However, such low-level verbal/phonological rehearsal appears to rely on lower-level speech processing mechanisms (e.g. Scott and Perrachione 2019) and the domain-general multiple-demand network (e.g. Fedorenko et al. 2011; Shashidhara et al. 2020), not on the language network. In any case, the verbal rehearsal account is quite different from L&M’s original LD-specific language recruitment hypothesis.

### Relationship to other work on language and categorization

Other results from psycho- and neurolinguistics also support the view that linguistic resources do not typically mediate categorization in humans. If access to linguistic representations were necessary for categorization, categorizing images would take longer than categorizing words; instead, they take approximately the same amount of time (Potter and Faulconer 1975). When asked to match a picture with a label, participants do not explicitly generate/rehearse verbal labels in advance unless there is an additional memory demand (e.g. if images disappear from the screen) (Pontillo et al. 2015). Previous work also shows that language is not necessary for performing tasks that require isolating a specific aspect (“feature”) of the semantic representation, including theory of mind inferences (Varley and Siegal 2000; Varley et al. 2001; Apperly et al. 2006) and thematic role identification (Ivanova et al. 2021). Our work therefore adds to the growing body of evidence for a separation between linguistic and visual semantic processing.

That said, many studies have shown that *linguistic labels* influence categorization behavior in infants (e.g. Gershkoff-Stowe et al. 1997; Sloutsky and Fisher 2004; Plunkett et al. 2008; Waxman and Gelman 2009; Ferguson and Waxman 2017) and adults (e.g. Lupyan et al. 2007; Lupyan 2009; Brojde et al. 2011; Zettersten and Lupyan 2020), so the relationship between words and categories is clearly an important one. What we are showing here is that the mechanisms responsible for language processing are not engaged during object categorization, nor are they specifically recruited for LD categorization. It is possible that linguistic labels, once acquired, may influence categorization via other brain systems, e.g. semantic, domain-general, or perceptual. The cognitive and neural mechanisms underlying the influence of labels on categorization thus remain to be determined (for some modeling proposals, see Gliozzi et al. 2009; Lupyan 2012; Ivanova and Hofer 2020; Luo et al. 2023).

Overall, our study shows that categorizing items is not a language-dependent task in the adult brain, regardless of whether the categorization is made on the basis of multiple features (HD) or a single feature (LD). Instead, this task relies on the domain-general multiple demand system, which supports diverse goal-directed behaviors. Our work provides evidence against the view of language as an aid for feature-based (LD) categorization and highlights the value of complementing patient studies with neuroimaging experiments.

## Supplementary Material

Appendix_1_final_bhad289

Appendix_2_final_bhad289

Appendix_3_final_bhad289

## CRediT taxonomy

Yael Benn (Conceptualization, Formal analysis, Methodology, Project administration, Software, Validation, Writing—original draft, Writing—review and editing), Anna Ivanova (Conceptualization, Data curation, Formal analysis, Validation, Visualization, Writing—original draft, Writing—review and editing), Oliver Clark (Formal analysis, Software, Validation), Zachary Mineroff (Formal analysis, Investigation, Methodology), Chloe Seikus (Investigation, Methodology), Jack Santos Silva (Investigation, Methodology), Rosmary Varley (Conceptualization, Investigation, Methodology, Supervision, Writing—original draft, Writing—review and editing), Evelina Fedorenko (Conceptualization, Methodology, Supervision, Writing—original draft, Writing—review and editing).
