# Supplementary material for: The language network is not engaged in object categorization
Source: Cereb Cortex. 2023 Aug 9;33(19):10380–400. doi: 10.1093/cercor/bhad289 (PMC10545444; doi:10.1093/cercor/bhad289)
Supplement: Appendix_1_final_bhad289 [file appendix_1_final_bhad289.docx]

**Appendix 1: Category Details**

***Table 1.*** *Categories used in all studies along with their dimension (HD=high-dimensional, LD=low-dimensional) and an alternative category type (semantic vs perceptual).*

| **Category** | **Dimension** | **Semantic vs. Perceptual** |
| --- | --- | --- |
| animals that live in water | HD | SEM |
| birds | HD | SEM |
| clothes | HD | SEM |
| dangerous animals | HD | SEM |
| farm animals | HD | SEM |
| fruit | HD | SEM |
| home appliances | HD | SEM |
| insects | HD | SEM |
| musical instruments | HD | SEM |
| non food things found in the kitchen | HD | SEM |
| objects found in the laundry room | HD | SEM |
| objects that hold water | HD | SEM |
| objects used for transportation | HD | SEM |
| things that fly | HD | SEM |
| tools | HD | SEM |
| vegetables | HD | SEM |
| animals with stripes | LD | PERC |
| long thin objects | LD | PERC |
| small objects | LD | SEM |
| things made of wood | LD | PERC |
| things that are blue | LD | PERC |
| things that are brown | LD | PERC |
| things that are green | LD | PERC |
| things that are orange | LD | PERC |
| things that are red | LD | PERC |
| things that are round | LD | PERC |
| things that are soft | LD | SEM |
| things that are very large | LD | SEM |
| things that are white | LD | PERC |
| things that are yellow | LD | PERC |
| things with doors | LD | SEM |
| things with handles | LD | SEM |

SEM – Semantic, PERC- Perceptual, HD- High Dimension, LD – Low Dimension

***Table 2.*** *Distribution of category use across participants (i.e., the number of times each participant saw each category during the fMRI categorization experiment, summed across runs).*

|  |  | **Subject ID** | | | | | | | | | | | | | |
| --- | --- | --- | --- | --- | --- | --- | --- | --- | --- | --- | --- | --- | --- | --- | --- |
| **Condition** | **Category** | **1** | **2** | **3** | **4** | **5** | **6** | **7** | **8** | **9** | **10** | **11** | **12** | **13** | **14** |
| HD | animals that live in water | 1 | 1 | 1 | 0 | 0 | 0 | 1 | 1 | 2 | 1 | 1 | 2 | 2 | 2 |
| HD | birds | 2 | 0 | 1 | 1 | 1 | 3 | 1 | 2 | 1 | 2 | 3 | 1 | 3 | 1 |
| HD | clothes | 1 | 1 | 1 | 1 | 1 | 0 | 1 | 0 | 0 | 2 | 1 | 0 | 1 | 1 |
| HD | dangerous animals | 2 | 1 | 2 | 1 | 1 | 0 | 1 | 1 | 1 | 1 | 1 | 0 | 0 | 1 |
| HD | farm animals | 1 | 1 | 3 | 2 | 0 | 0 | 2 | 2 | 2 | 1 | 3 | 1 | 2 | 1 |
| HD | fruit | 1 | 2 | 1 | 1 | 2 | 2 | 0 | 0 | 1 | 1 | 1 | 2 | 1 | 0 |
| HD | home appliances | 2 | 2 | 0 | 1 | 1 | 2 | 2 | 1 | 0 | 1 | 0 | 2 | 0 | 1 |
| HD | insects | 2 | 2 | 1 | 3 | 1 | 2 | 1 | 2 | 1 | 2 | 1 | 2 | 1 | 2 |
| HD | musical instruments | 0 | 1 | 1 | 0 | 3 | 0 | 2 | 1 | 2 | 2 | 2 | 1 | 1 | 1 |
| HD | non food things found in the kitchen | 2 | 1 | 0 | 0 | 2 | 2 | 0 | 2 | 2 | 1 | 1 | 2 | 0 | 1 |
| HD | objects found in the laundry room | 1 | 1 | 0 | 2 | 1 | 1 | 1 | 2 | 1 | 1 | 1 | 0 | 1 | 1 |
| HD | objects that hold water | 0 | 1 | 2 | 2 | 0 | 1 | 2 | 0 | 0 | 1 | 1 | 1 | 2 | 2 |
| HD | objects used for transportation | 1 | 2 | 1 | 1 | 1 | 1 | 0 | 1 | 3 | 1 | 0 | 0 | 1 | 1 |
| HD | things that fly | 1 | 2 | 3 | 1 | 0 | 1 | 2 | 2 | 1 | 0 | 1 | 2 | 0 | 2 |
| HD | tools | 1 | 0 | 0 | 2 | 2 | 2 | 2 | 0 | 0 | 0 | 0 | 1 | 0 | 0 |
| HD | vegetables | 0 | 0 | 1 | 0 | 2 | 1 | 0 | 1 | 1 | 1 | 1 | 1 | 3 | 1 |
| LD | animals with stripes | 2 | 1 | 2 | 2 | 0 | 1 | 2 | 0 | 1 | 2 | 0 | 1 | 0 | 2 |
| LD | long thin objects | 0 | 0 | 2 | 2 | 1 | 1 | 1 | 1 | 0 | 2 | 2 | 1 | 2 | 2 |
| LD | small objects | 1 | 2 | 0 | 1 | 2 | 1 | 0 | 1 | 1 | 1 | 1 | 0 | 1 | 1 |
| LD | things made of wood | 1 | 1 | 1 | 3 | 2 | 1 | 2 | 1 | 0 | 1 | 2 | 2 | 0 | 0 |
| LD | things that are blue | 2 | 1 | 1 | 1 | 1 | 1 | 1 | 1 | 0 | 0 | 1 | 0 | 1 | 1 |
| LD | things that are brown | 1 | 2 | 1 | 0 | 3 | 1 | 2 | 2 | 2 | 1 | 2 | 0 | 1 | 1 |
| LD | things that are green | 1 | 0 | 1 | 1 | 0 | 2 | 1 | 1 | 2 | 2 | 1 | 1 | 0 | 1 |
| LD | things that are orange | 2 | 1 | 2 | 2 | 1 | 2 | 0 | 1 | 1 | 1 | 0 | 2 | 2 | 1 |
| LD | things that are red | 1 | 1 | 1 | 1 | 0 | 1 | 1 | 1 | 2 | 0 | 2 | 1 | 2 | 1 |
| LD | things that are round | 1 | 1 | 1 | 1 | 1 | 1 | 0 | 1 | 3 | 1 | 2 | 0 | 1 | 1 |
| LD | things that are soft | 0 | 0 | 2 | 0 | 2 | 1 | 1 | 2 | 1 | 0 | 2 | 2 | 1 | 2 |
| LD | things that are very large | 1 | 1 | 1 | 2 | 1 | 2 | 1 | 1 | 1 | 1 | 1 | 2 | 3 | 0 |
| LD | things that are white | 3 | 2 | 1 | 1 | 1 | 0 | 1 | 0 | 1 | 1 | 0 | 3 | 0 | 2 |
| LD | things that are yellow | 2 | 3 | 0 | 0 | 0 | 1 | 2 | 2 | 1 | 3 | 1 | 1 | 2 | 0 |
| LD | things with doors | 0 | 1 | 1 | 0 | 1 | 1 | 2 | 2 | 1 | 1 | 0 | 1 | 2 | 3 |
| LD | things with handles | 0 | 1 | 1 | 1 | 2 | 1 | 1 | 1 | 1 | 1 | 1 | 1 | 0 | 0 |
