# Supplementary material for: The language network is not engaged in object categorization
Source: Cereb Cortex. 2023 Aug 9;33(19):10380–400. doi: 10.1093/cercor/bhad289 (PMC10545444; doi:10.1093/cercor/bhad289)
Supplement: Appendix_2_final_bhad289 [file appendix_2_final_bhad289.docx]

# Appendix 2: Additional analyses for Studies 1 & 2

## Hit Rate and False Alarm Results

***Study 1***

***Hit rate***. Participants with aphasia had similar hit rates for LD categories (*M*=0.84, *SD*=0.07) and HD categories (*M*=0.89, *SD*=0.07; LD>HD: *β*=-0.41, *SE*=0.28, *p*=.139). The overall hit rate for participants with aphasia (*M*=0.87, *SD*=0.08) was similar to neurotypical participants (*M*=0.90, *SD*=0.06; neurotypical>aphasia: *β*=0.24, *SE*=0.25, *p*=.338) and lower than for participants with PD (*M*=0.93, *SD*=0.03; PD>aphasia: *β*=0.72, *SE*=0.23, *p*=.002). Moreover, we did not observe a reliable category dimension by group interaction for the aphasia vs. neurotypical comparison (*β*=0.04, *SE*=0.18, *p*=.813), nor for the aphasia vs. PD comparison (*β*=0.19, *SE*=0.18, *p*=.304). Follow-up analyses showed that there was no main effect of category dimension across groups (*β*=0.34, *SE*=0.27, *p*=.813), nor within the neurotypical group (*β*=0.37, *SE*=0.29, *p*=.478) or the PD group (*β*=0.22, *SE*=0.29, *p*=.788). These results fail to replicate the findings by L&M, who reported the main effect of category dimension, as well as a selective impairment in LD categorization for patients with aphasia.

We additionally conducted an exploratory analysis to investigate the difference between the aphasia and PD groups. Given that the PD group had a higher average education level, we repeated the analysis above with ‘education level’ as an additional fixed effect. The updated model had a similar fit to the data compared to the original (as per the likelihood ratio test: χ^2^=3.39, *p*=.065); under this model, the difference between the aphasia and the PD groups was no longer significant (*β*=0.37, *SE*=0.29, *p*=.202). The significance of other effects was unchanged.

***False alarm rate***. The false alarm rate in participants with aphasia also did not differ between LD categories (*M*=0.03, *SD*=0.03) and HD categories (*M*=0.03, *SD*=0.03; LD>HD: *β*=-0.22, *SE*=0.35, *p*=.534). As with the hit rate, the overall false alarm rate for participants with aphasia (*M*=0.03, *SD*=0.03) was comparable to that of neurotypical participants (*M*=0.01, *SD*=0.01; neurotypical>aphasia: *β*=-0.58, *SE*=0.37, *p*=.123), although participants with PD performed better than participants with aphasia, i.e., with fewer false alarms (*M*=0.01, *SD*=0.01; PD>aphasia: *β*=-0.74, *SE*=.34, *p*=.031). Unlike the hit rate results above, there was a significant interaction between category dimension (LD>HD) and group (neurotypical>aphasia: *β*=0.63, *SE*=0.23, *p*=.006; PD>aphasia: *β*=0.44, *SE*=0.21, *p*=.034). However, this interaction effect goes in the opposite direction from that predicted by the LD-specific language recruitment hypothesis: participants with aphasia performed *better* on LD categories relative to controls. The pattern of results is also inconsistent with L&M’s results in that they found no interaction between group and category dimension. Lastly, follow-up analyses showed no main effect of category dimension across groups (*β*=-0.14, *SE*=0.34, *p*=.951), nor within the neurotypical group (*β*=-0.41, *SE*=0.38, *p*=.614) or the PD group (*β*=-0.22, *SE*=0.37, *p*=.879).

Similar to the hit rate analysis, an exploratory model that included ‘education level’ as a fixed effect explained a similar amount of variance compared to the original model (χ^2^=0.35, *p*=.557) and no longer showed a significant difference between the aphasia and PD groups (β=-0.57, *SE*=0.45, *p*=.209). The significance of other effects was unchanged.


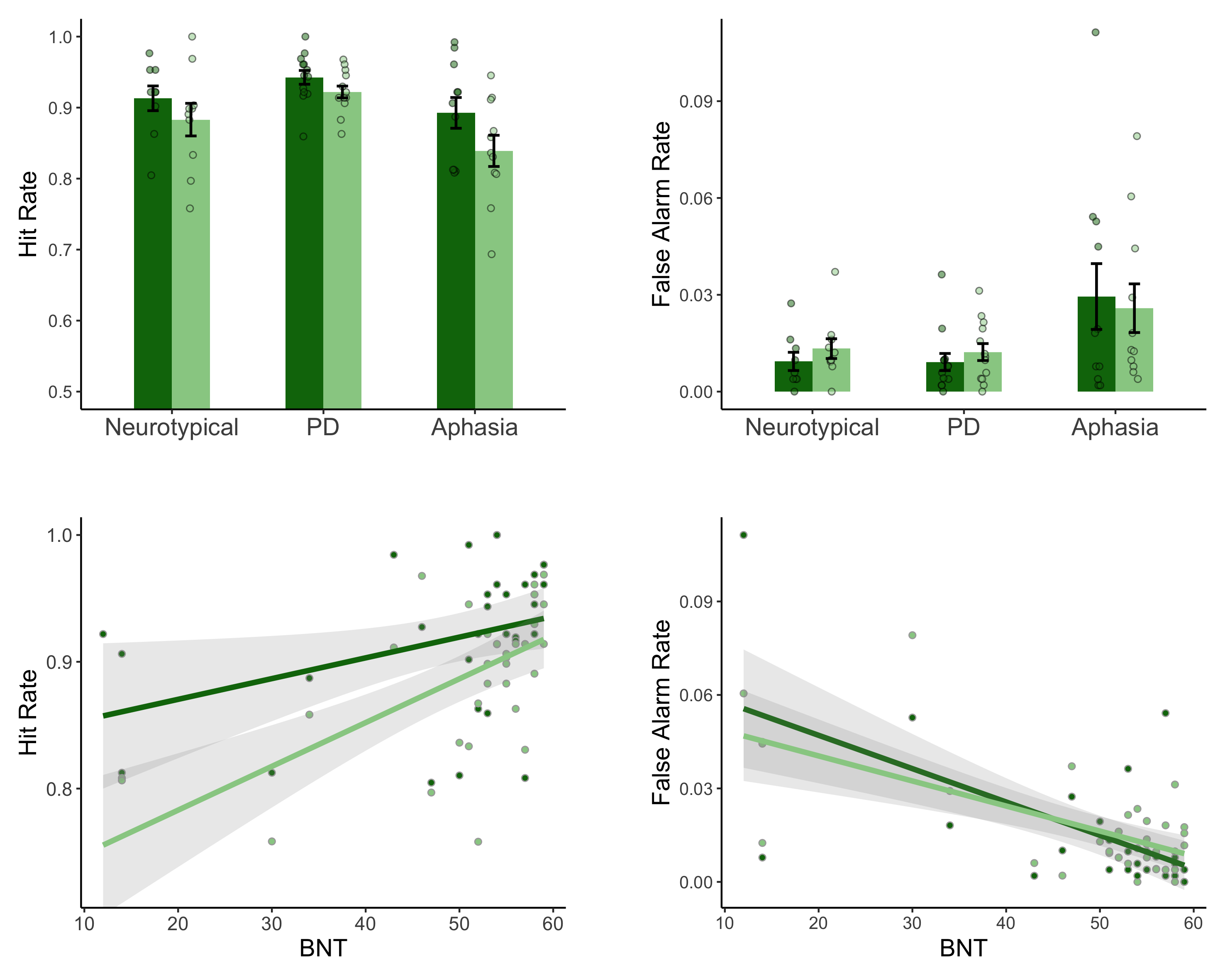


***Figure 1****. Study 1 results. Top: Hit Rate (left) and False Alarm Rate (right) across the three participant groups. Bottom: Hit Rate (left) and False Alarm Rate (right) plotted against participants’ BNT scores, a measure of naming performance.*

***Study 2***

***Hit rate***. Similar to the results of Study 1, participants with aphasia had similar hit rates for LD categories (*M*=0.88, *SD*=0.08) and HD categories (*M*=0.91, *SD*=0.06; LD>HD: *β*=-0.34, *SE*=0.29, *p*=.252). Participants with aphasia had overall lower hit rates (*M*=0.90, *SD*=0.07) compared to neurotypical participants (*M*=0.97, *SD*=0.02; neurotypical>aphasia: *β*=1.45, *SE*=0.32, *p*<.001) and participants with PD (*M*=0.95, *SD*=0.03; PD>aphasia: *β*=0.86, *SE*=0.31, *p*=.005), which is consistent with Study 1’s negative relationship between naming ability and categorization performance. We did not observe a reliable category dimension by group interaction for the aphasia vs. neurotypical comparison (*β*=0.44, *SE*=0.26, *p*=.086), nor for the aphasia vs. PD comparison (*β*=0.42, *SE*=0.23, *p*=.070). Follow-up analysis showed that there was no main effect of category dimension across groups (*β*=0.05, *SE*=0.25, *p*=.990), nor within the neurotypical group (*β*=-0.11, *SE*=0.30, *p*=.960), or the PD group (*β*=-0.08, *SE*=0.28, *p*=.975). Overall, the group comparison of hit rate does not support the LD-specific language recruitment hypothesis.

***False alarm rate***. The false alarm rate results (**Figure 3B**) were consistent with the hit rate results. Participants with aphasia had comparable false alarm rates for LD categories (*M*=0.13, *SD*=0.08) and HD categories (*M*=0.11, *SD*=0.10; LD>HD: *β*=0.13, *SE*=0.27, *p*=.626). The overall false alarm rate among participants with aphasia (*M*=0.12, *SD*=0.09) was higher than in neurotypical participants (*M*=0.02, *SD*=0.02; neurotypical>aphasia: *β*=-1.91, *SE*=0.32, *p*<.001) and participants with PD (*M*=0.02, *SD*=0.02; PD>aphasia: *β*=-1.94, *SE*=0.32, *p*<.001). The group by category dimension interactions were not significant for either the neurotypical vs. aphasia comparison (*β*=-.38, *SE*=.21, *p*=.075), nor the PD vs. aphasia comparison (*β*=-0.19, *SE*=0.22, *p*=.381). Follow-up analyses showed no effect of category dimension across groups (*β*=0.06, *SE*=0.25, *p*=.985), nor within the neurotypical group (*β*=0.25, *SE*=0.29, *p*=.732) or the PD group (*β*=0.06, *SE*=0.29, *p*=.990).


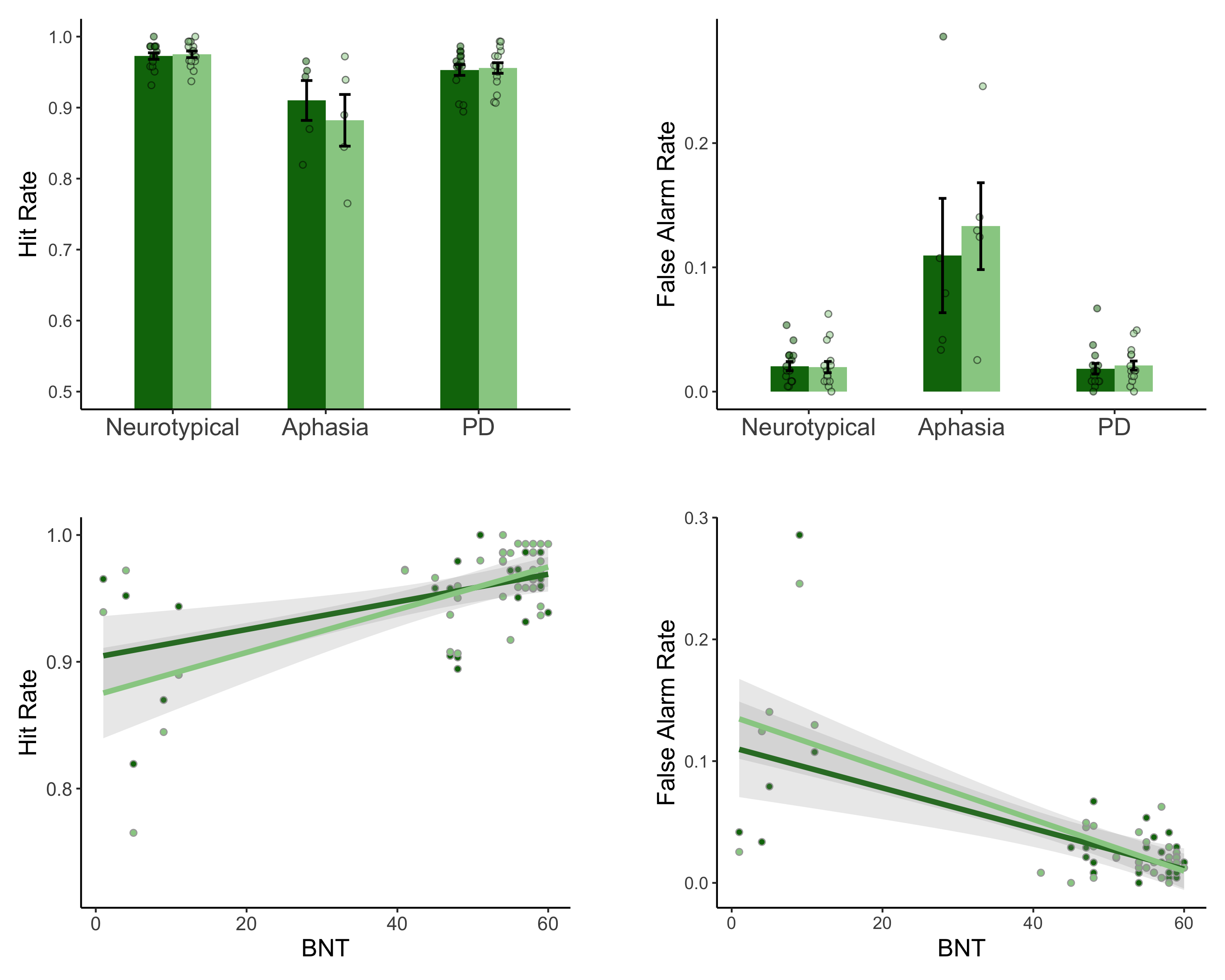


***Figure 2****. Study 2 results. Top: Hit Rate (left) and False Alarm Rate (right) across the three participant groups. Bottom: Hit Rate (left) and False Alarm Rate (right) plotted against participants’ BNT scores, a measure of naming performance.*

## Semantic vs. perceptual categories

***Study 1***

There was no main effect of category type (semantic vs. perceptual) on accuracy, nor was there an interaction between category type and participant group. The only significant effect was the effect of PD group (PD > aphasia; *β*=0.65, *SE*=0.24, *p*=.007). As mentioned in the main text, this difference is likely caused by the higher education level in the PD; indeed, the effect is no longer significant once education is included as a covariate (the two models have similar predictive power as estimated by the likelihood ratio test).

Response times varied as an effect of group (neurotypical > aphasia: *β*=-8.18, *SE*=4.02, *p*=.05; PD > aphasia: *β*=-12.7, *SE*=3.66, *p*=.001), category type (semantic > perceptual: *β*=9.71, *SE*=2.08, *p*<.001), and an interaction between category type and PD vs. aphasia groups (*β*=-4.31, *SE*=1.23, *p*<.001). When education was included as covariate, the main effect of PD>aphasia group was no longer significant, but other effects remained. Thus, semantic categories are overall more challenging than perceptual categories for all groups, but the difference is greater for the aphasia group than for the PD group (one of the two controls).

The BNT analyses showed no effects of BNT on accuracy; however, BNT was a significant predictor of response times (*β*=-4.19, *SE*=1.41, *p*=.001) and interacted significantly with category dimension (*β*=-2.20, *SE*=.52, *p*<.001), such that the semantic-perceptual response time gap increased if BNT was low.


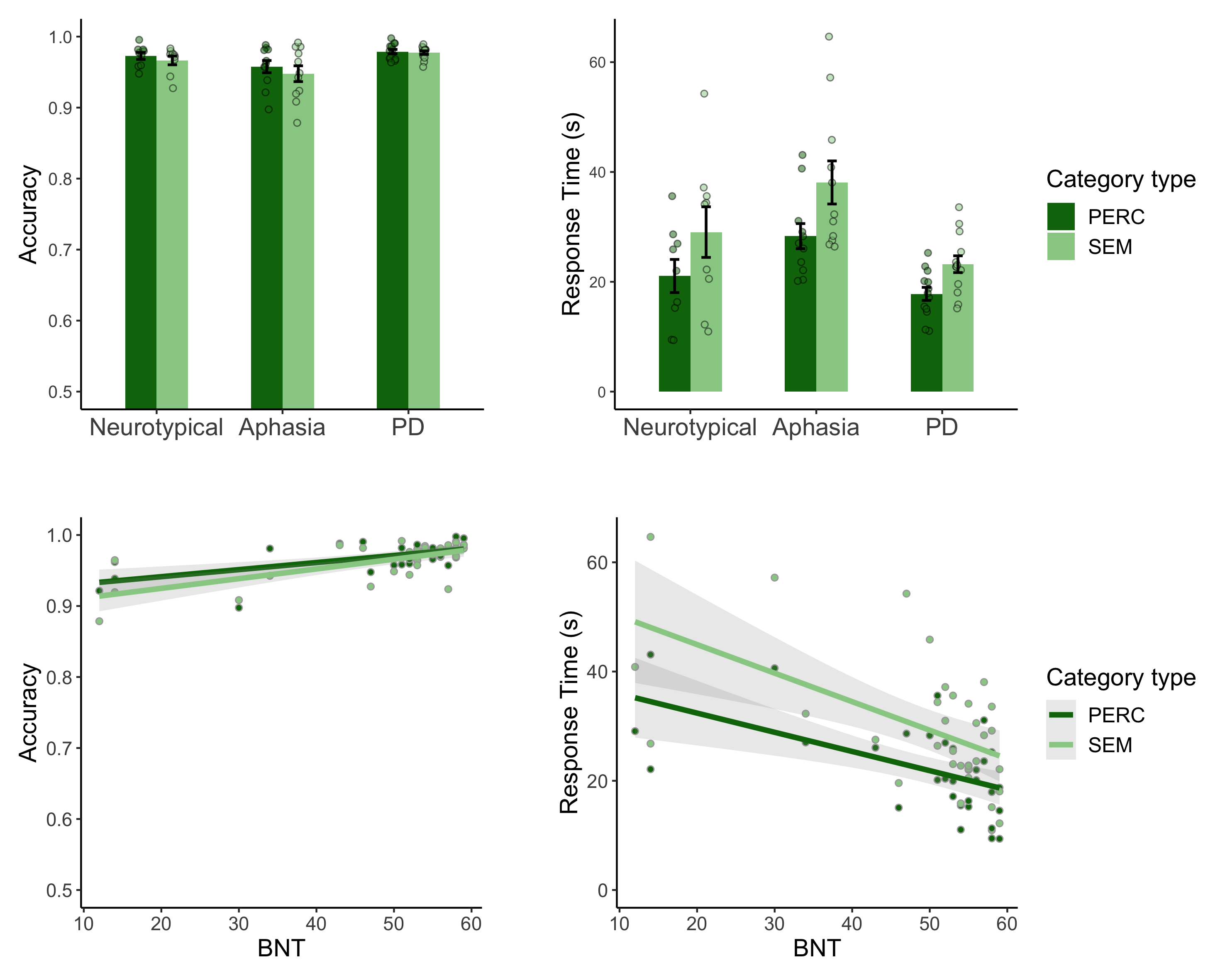


***Figure 3****. Study 1 results. Top: Accuracy (left) and Response Time (RT; right) across the three participant groups (here, RT is the time until participants pressed a “yes” or “no” button for each image within a trial). Bottom: Accuracy (left) and RT (right)plotted against participants’ BNT scores, a measure of naming performance.*

***Study 2***

Participants with aphasia performed overall worse than neurotypical controls (*β*=1.74, *SE*=.28, *p*<.001) and than participants with PD (*β*=1.48, *SE*=.28, *p*<.001). However, there was no main effect of category type nor an interaction between category type and participant group. BNT was a significant predictor of accuracy (*β*=.51, *SE*=.11, *p*<.001), but did not interact with category type.

Response times analysis showed the main effect of group (neurotypical > aphasia: *β*=-.84, *SE*=.19, *p*<.001; PD > aphasia: *β*=-.88, *SE*=1.87, *p*<.001), no main effect of category type, and an interaction between category type and participant group (neurotypical > aphasia: *β*=.18, *SE*=.04, *p*<.001; PD > aphasia: *β*=.14, *SE*=.04, *p*<.001). The interaction effect goes in the opposite direction from that predicted by the initial hypothesis. The BNT analysis is consistent with the group analysis: BNT is a significant predictor of response time (*β*=.30, *SE*=.07, *p*<.001), and there is an interaction between BNT and category type (*β*=.06, *SE*=.01, *p*<.001), such that participants with high BNT are faster at perceptual categories, but those with low BNT are not.


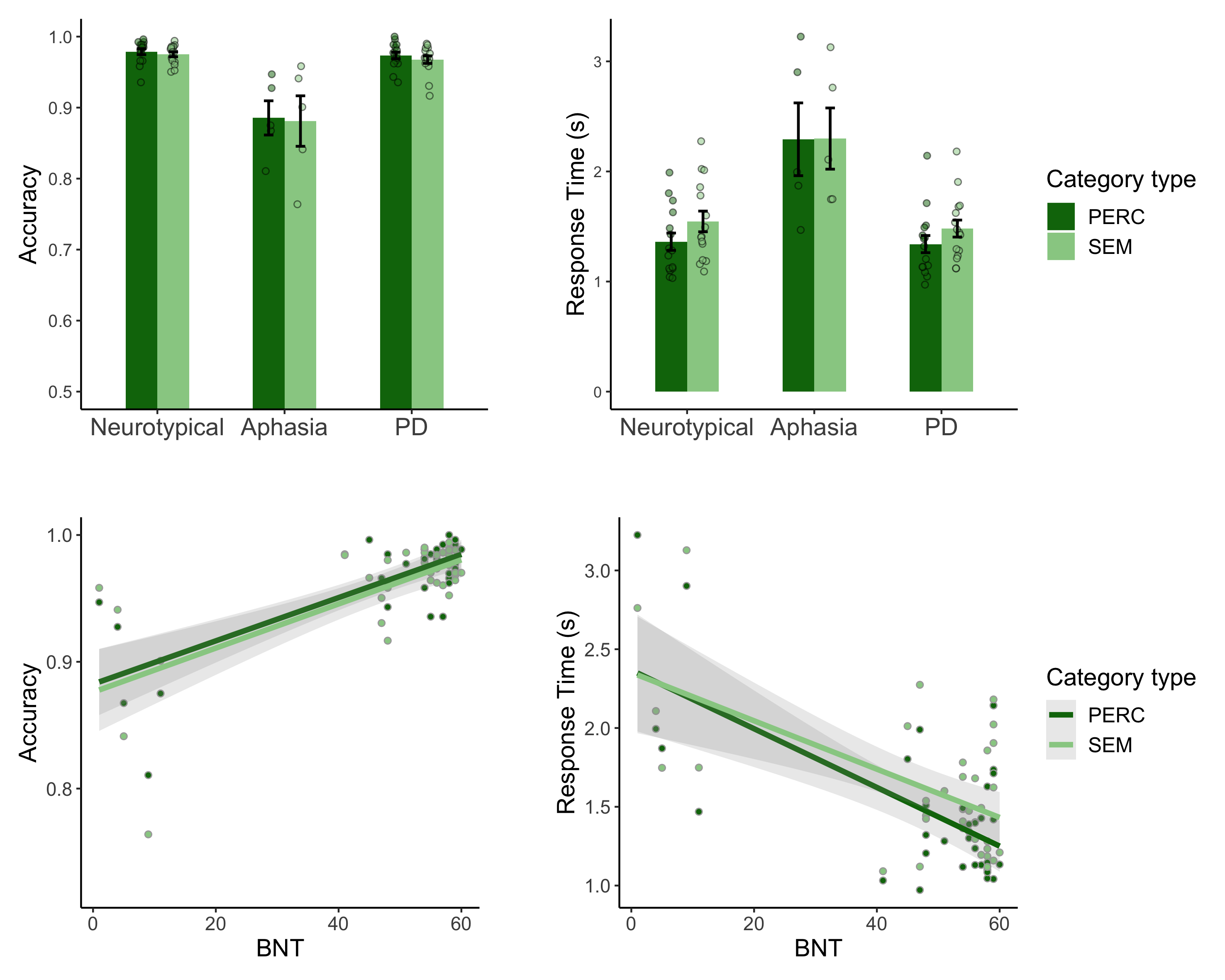


***Figure 4****. Study 2 results. Top: Accuracy (left) and Response Time (RT; right) across the three participant groups (here, RT is the time until participants pressed a “yes” or “no” button for each image within a trial). Bottom: Accuracy (left) and RT (right)plotted against participants’ BNT scores, a measure of naming performance.*
