# Supplementary material for: The language network is not engaged in object categorization
Source: Cereb Cortex. 2023 Aug 9;33(19):10380–400. doi: 10.1093/cercor/bhad289 (PMC10545444; doi:10.1093/cercor/bhad289)
Supplement: Appendix_3_final_bhad289 [file appendix_3_final_bhad289.docx]

# Appendix 3: Additional information & analyses, fMRI experiment

1. **Statistics details**

Condition contrasts were designed to test the following null hypotheses.

Language network:

1. $\frac{HD+LD}{2}=0$
2. $LD=HD$ (main)
3. $\frac{HD+LD2}{2}=Sentences$
4. $\frac{HD+LD}{2}=Nonwords$

LD – low-dimensional categorization; HD – high-dimensional categorization.

Multiple demand network:

1. $\frac{HD+LD}{2}=0$
2. $LD=HD$ (main)
3. $HardWM=EasyWM$
4. $\frac{HD+LD}{2}=\frac{HardWM+EasyWM}{2}$
5. $\frac{HD+LD}{2}=Sentences$
6. $\frac{HD+LD}{2}=Nonwords$

HardWM – hard working memory task, EasyWM – easy working memory task.

For semantic vs. perceptual analyses, the analyses were identical except “SEM” and “PERC” category labels were used instead of “HD” and “LD”.

Putative LD categorization regions (results reported in the main text):

1. $\frac{HD+LD}{2}=0$
2. $LD=HD$ (main)
3. $HardWM=EasyWM$
4. $Sentences=Nonwords$
5. $\frac{HD+LD}{2}=\frac{HardWM+EasyWM}{2}$
6. $\frac{HD+LD}{2}=Nonwords$
7. **Results for individual fROIs**

***Table 1****. Mixed-effect linear regression results for language fROIs. p-values were FDR-corrected for the number of fROIs. Significant p-values are highlighted in bold. S – sentence reading, N – nonword reading, LD – low-dimensional categorization, HD – high-dimensional categorization.*

| **ROI** | **Regression Term** | ***Beta*** | ***p-*value** |
| --- | --- | --- | --- |
| IFGorb | **Categorization>0** | **0.48** | **.005** |
|  | LD>HD | 0.04 | .932 |
|  | **S>Categorization** | **1.21** | **<.001** |
|  | N>Categorization | 0.02 | .905 |
| IFG | **Categorization>0** | **0.90** | **<.001** |
|  | LD>HD | 0.02 | .932 |
|  | **S>Categorization** | **1.41** | **<.001** |
|  | N>Categorization | -0.15 | .510 |
| MFG | **Categorization>0** | **0.63** | **.005** |
|  | LD>HD | 0.11 | .932 |
|  | **S>Categorization** | **2.41** | **<.001** |
|  | **N>Categorization** | **0.75** | **.031** |
| PostTemp | Categorization>0 | 0.27 | .094 |
|  | LD>HD | 0.10 | .932 |
|  | **S>Categorization** | **1.81** | **<.001** |
|  | N>Categorization | 0.26 | .114 |
| AntTemp | Categorization>0 | -0.02 | .760 |
|  | LD>HD | -0.08 | .932 |
|  | **S>Categorization** | **1.52** | **<.001** |
|  | N>Categorization | 0.22 | .114 |
| AngG | Categorization>0 | 0.24 | .165 |
|  | LD>HD | -0.30 | .575 |
|  | **S>Categorization** | **0.57** | **<.001** |
|  | N>Categorization | -0.34 | .086 |

***Table 2****. Mixed-effect linear regression results for multiple demand fROIs. P-values were FDR-corrected for the number of fROIs. Significant p-values are highlighted in bold. H – hard working memory task, E – easy working memory task, LD – low-dimensional categorization, HD – high-dimensional categorization.*

| **Hemisphere** | **fROI #** | **fROI name** | **Regression Term** | ***Beta*** | ***p*-value** |
| --- | --- | --- | --- | --- | --- |
| L | 1 | postParietal | **Categorization>0** | **1.44** | **<.001** |
|  |  |  | LD>HD | 0.28 | .902 |
|  |  |  | **Hard WM>Easy WM** | **1.37** | **<.001** |
|  |  |  | **WM>Categorization** | **2.69** | **<.001** |
|  |  |  | **Nonwords>Categorization** | **-0.90** | **<.001** |
|  |  |  | **Sentences>Categorization** | **-1.36** | **<.001** |
| L | 2 | midParietal | **Categorization>0** | **0.98** | **.004** |
|  |  |  | LD>HD | 0.30 | .902 |
|  |  |  | **Hard WM>Easy WM** | **1.40** | **<.001** |
|  |  |  | **WM>Categorization** | **2.32** | **<.001** |
|  |  |  | Nonwords>Categorization | -0.07 | .913 |
|  |  |  | **Sentences>Categorization** | **-0.65** | **.034** |
| L | 3 | antParietal | **Categorization>0** | **0.88** | **.003** |
|  |  |  | LD>HD | 0.47 | .902 |
|  |  |  | **Hard WM>Easy WM** | **1.14** | **<.001** |
|  |  |  | **WM>Categorization** | **2.10** | **<.001** |
|  |  |  | Nonwords>Categorization | -0.17 | .567 |
|  |  |  | **Sentences>Categorization** | **-0.71** | **.004** |
| L | 4 | supFrontal | **Categorization>0** | **0.77** | **.007** |
|  |  |  | LD>HD | 0.23 | .902 |
|  |  |  | **Hard WM>Easy WM** | **0.97** | **.002** |
|  |  |  | **WM>Categorization** | **2.16** | **<.001** |
|  |  |  | Nonwords>Categorization | -0.28 | .355 |
|  |  |  | Sentences>Categorization | -0.38 | .139 |
| L | 5 | precentral_A | **Categorization>0** | **2.23** | **<.001** |
|  |  |  | LD>HD | 0.37 | .902 |
|  |  |  | **Hard WM>Easy WM** | **1.34** | **<.001** |
|  |  |  | **WM>Categorization** | **0.98** | **<.001** |
|  |  |  | Nonwords>Categorization | -0.56 | .058 |
|  |  |  | **Sentences>Categorization** | **-0.97** | **<.001** |
| L | 6 | precentral_B | **Categorization>0** | **1.42** | **<.001** |
|  |  |  | LD>HD | 0.17 | .902 |
|  |  |  | **Hard WM>Easy WM** | **1.12** | **<.001** |
|  |  |  | **WM>Categorization** | **0.71** | **.001** |
|  |  |  | **Nonwords>Categorization** | **-0.66** | **.030** |
|  |  |  | **Sentences>Categorization** | **-0.77** | **.004** |
| L | 7 | midFrontal | **Categorization>0** | **1.29** | **<.001** |
|  |  |  | LD>HD | 0.18 | .902 |
|  |  |  | **Hard WM>Easy WM** | **0.94** | **<.001** |
|  |  |  | WM>Categorization | 0.25 | .178 |
|  |  |  | **Nonwords>Categorization** | **-0.86** | **.002** |
|  |  |  | **Sentences>Categorization** | **-1.25** | **<.001** |
| L | 8 | midFrontalOrb | **Categorization>0** | **1.15** | **.006** |
|  |  |  | LD>HD | 0.13 | .902 |
|  |  |  | **Hard WM>Easy WM** | **1.29** | **<.001** |
|  |  |  | **WM>Categorization** | **0.65** | **.003** |
|  |  |  | **Nonwords>Categorization** | **-0.62** | **.034** |
|  |  |  | **Sentences>Categorization** | **-1.08** | **<.001** |
| L | 9 | insula | **Categorization>0** | **0.81** | **<.001** |
|  |  |  | LD>HD | -0.01 | .953 |
|  |  |  | **Hard WM>Easy WM** | **0.72** | **<.001** |
|  |  |  | **WM>Categorization** | **0.49** | **<.001** |
|  |  |  | **Nonwords>Categorization** | **-0.38** | **.008** |
|  |  |  | **Sentences>Categorization** | **-0.49** | **<.001** |
| L | 10 | medialFrontal | **Categorization>0** | **0.95** | **<.001** |
|  |  |  | LD>HD | -0.01 | .953 |
|  |  |  | **Hard WM>Easy WM** | **0.79** | **<.001** |
|  |  |  | **WM>Categorization** | **0.59** | **<.001** |
|  |  |  | **Nonwords>Categorization** | **-0.40** | **.033** |
|  |  |  | **Sentences>Categorization** | **-0.56** | **.001** |
| R | 1 | postParietal | **Categorization>0** | **1.15** | **<.001** |
|  |  |  | LD>HD | 0.26 | .902 |
|  |  |  | **Hard WM>Easy WM** | **1.68** | **<.001** |
|  |  |  | **WM>Categorization** | **3.18** | **<.001** |
|  |  |  | **Nonwords>Categorization** | **-0.84** | **.009** |
|  |  |  | **Sentences>Categorization** | **-1.14** | **<.001** |
| R | 2 | midParietal | **Categorization>0** | **0.74** | **.004** |
|  |  |  | LD>HD | 0.31 | .902 |
|  |  |  | **Hard WM>Easy WM** | **1.72** | **<.001** |
|  |  |  | **WM>Categorization** | **2.09** | **<.001** |
|  |  |  | Nonwords>Categorization | 0.05 | .913 |
|  |  |  | Sentences>Categorization | -0.43 | .148 |
| R | 3 | antParietal | **Categorization>0** | **0.33** | **.040** |
|  |  |  | LD>HD | 0.35 | .902 |
|  |  |  | **Hard WM>Easy WM** | **1.23** | **<.001** |
|  |  |  | **WM>Categorization** | **1.98** | **<.001** |
|  |  |  | Nonwords>Categorization | 0.00 | .989 |
|  |  |  | Sentences>Categorization | -0.27 | .273 |
| R | 4 | supFrontal | **Categorization>0** | **0.70** | **.013** |
|  |  |  | LD>HD | 0.14 | .902 |
|  |  |  | **Hard WM>Easy WM** | **1.55** | **<.001** |
|  |  |  | **WM>Categorization** | **2.77** | **<.001** |
|  |  |  | Nonwords>Categorization | -0.06 | .913 |
|  |  |  | Sentences>Categorization | -0.20 | .526 |
| R | 5 | precentral_A | **Categorization>0** | **1.48** | **<.001** |
|  |  |  | LD>HD | 0.19 | .902 |
|  |  |  | **Hard WM>Easy WM** | **1.40** | **<.001** |
|  |  |  | **WM>Categorization** | **1.22** | **<.001** |
|  |  |  | Nonwords>Categorization | -0.35 | .353 |
|  |  |  | **Sentences>Categorization** | **-0.68** | **.032** |
| R | 6 | precentral_B | **Categorization>0** | **1.73** | **<.001** |
|  |  |  | LD>HD | 0.29 | .902 |
|  |  |  | **Hard WM>Easy WM** | **1.65** | **<.001** |
|  |  |  | **WM>Categorization** | **1.21** | **<.001** |
|  |  |  | Nonwords>Categorization | -0.72 | .067 |
|  |  |  | **Sentences>Categorization** | **-1.08** | **.004** |
| R | 7 | midFrontal | **Categorization>0** | **0.91** | **.009** |
|  |  |  | LD>HD | 0.17 | .902 |
|  |  |  | **Hard WM>Easy WM** | **1.71** | **<.001** |
|  |  |  | **WM>Categorization** | **1.08** | **<.001** |
|  |  |  | Nonwords>Categorization | -0.26 | .485 |
|  |  |  | **Sentences>Categorization** | **-0.64** | **.036** |
| R | 8 | midFrontalOrb | **Categorization>0** | **0.89** | **.003** |
|  |  |  | LD>HD | 0.05 | .953 |
|  |  |  | **Hard WM>Easy WM** | **1.89** | **<.001** |
|  |  |  | **WM>Categorization** | **0.96** | **<.001** |
|  |  |  | Nonwords>Categorization | -0.39 | .306 |
|  |  |  | **Sentences>Categorization** | **-0.86** | **.007** |
| R | 9 | insula | **Categorization>0** | **0.72** | **.001** |
|  |  |  | LD>HD | -0.04 | .902 |
|  |  |  | **Hard WM>Easy WM** | **0.85** | **<.001** |
|  |  |  | **WM>Categorization** | **0.42** | **<.001** |
|  |  |  | **Nonwords>Categorization** | **-0.34** | **.013** |
|  |  |  | **Sentences>Categorization** | **-0.46** | **<.001** |
| R | 10 | medialFrontal | **Categorization>0** | **0.84** | **<.001** |
|  |  |  | LD>HD | 0.06 | .902 |
|  |  |  | **Hard WM>Easy WM** | **1.23** | **<.001** |
|  |  |  | **WM>Categorization** | **0.63** | **<.001** |
|  |  |  | Nonwords>Categorization | -0.35 | .073 |
|  |  |  | **Sentences>Categorization** | **-0.60** | **.002** |

1. **Results for semantic vs. perceptual comparison (with instruction reading)**

Response to semantic categories was not significantly different from response to perceptual categories (*β*=0.10, *SE*=0.09, *p*=.272) or nonword reading (*β*=0.08, *SE*=0.09, *p*=.385), and significantly weaker than responses to sentences (*β*=1.01, *SE*=.07, *p*<.001) and instruction reading (*β*=1.02, *SE*=.09, *p*<.001). Follow-up analyses in individual language fROIs showed that none of them had significantly different responses to semantic and perceptual categories. Four fROIs (lMFG, lIFG, lIFGorb, and rIFG) had above-zero responses to semantic categorization, but these responses were not significantly different from responses during the control nonword reading task. Thus, our results suggest that the language network is not involved in either semantic or perceptual categorization in neurotypical participants.


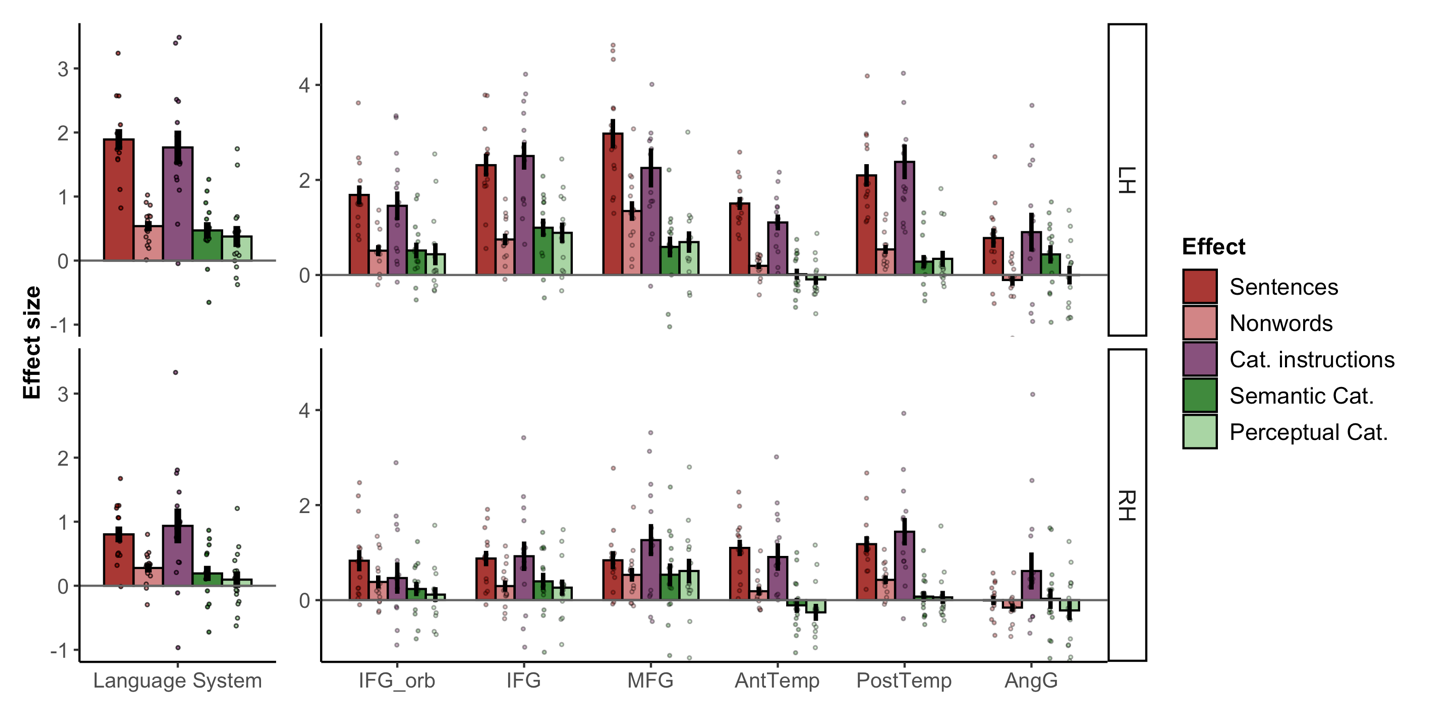


***Figure 1.*** *Categorization responses within the language brain network. Top: left hemisphere (LH); bottom: right hemisphere (RH). Left: responses averaged across fROIs; right: responses in individual fROIs. Abbreviations: Cat. – categorization, IFG – inferior frontal gyrus, orb – pars orbitalis, MFG – middle frontal gyrus, AntTemp – anterior temporal lobe, PostTemp – posterior temporal lobe, AngG – angular gyrus. Here and elsewhere, dots show values for individual participants, and error bars show standard error of the mean.*

The multiple demand network had equally strong responses to semantic and perceptual categories (*β*=0.14, *SE*=0.09, *p*=.139), which were significantly above 0 (*β*=1.16, *SE*=0.22, *p*<.001). Responses to semantic categorization were stronger than responses to control conditions from the language localizer task (semantic categorization > sentences: *β*=0.73, *SE*=0.09, *p*<.001; semantic categorization > nonwords: *β*=0.41, *SE*=0.09, *p*<.001). However, they were weaker than responses to the spatial working memory task (*β*=-1.41, *SE*=.08, *p*<.001), indicating that the working memory task was more effortful. They were also weaker than responses to the instruction stage of the categorization task (*β*=-0.64, *SE*=.09, *p*<.001), indicating that the most cognitively demanding stage of the task is the initial category label processing. The responses to categorization were stronger in the left hemisphere (*β*=0.26, *SE*=0.09, *p*=.005), but there was no interaction between hemisphere and category type (*β*=0.02, *SE*=0.18, *p*=.895). Follow-up analyses in individual fROIs showed that no fROIs had a significant difference between semantic and perceptual categorization. Responses to categorization overall were significantly above 0 in all fROIs, but weaker than the overall responses to the working memory task in almost all fROIs (except left middle frontal and middle frontal orbital fROIs). Thus, the MD network was engaged in categorization but did not show a preference for either semantic or perceptual categories.


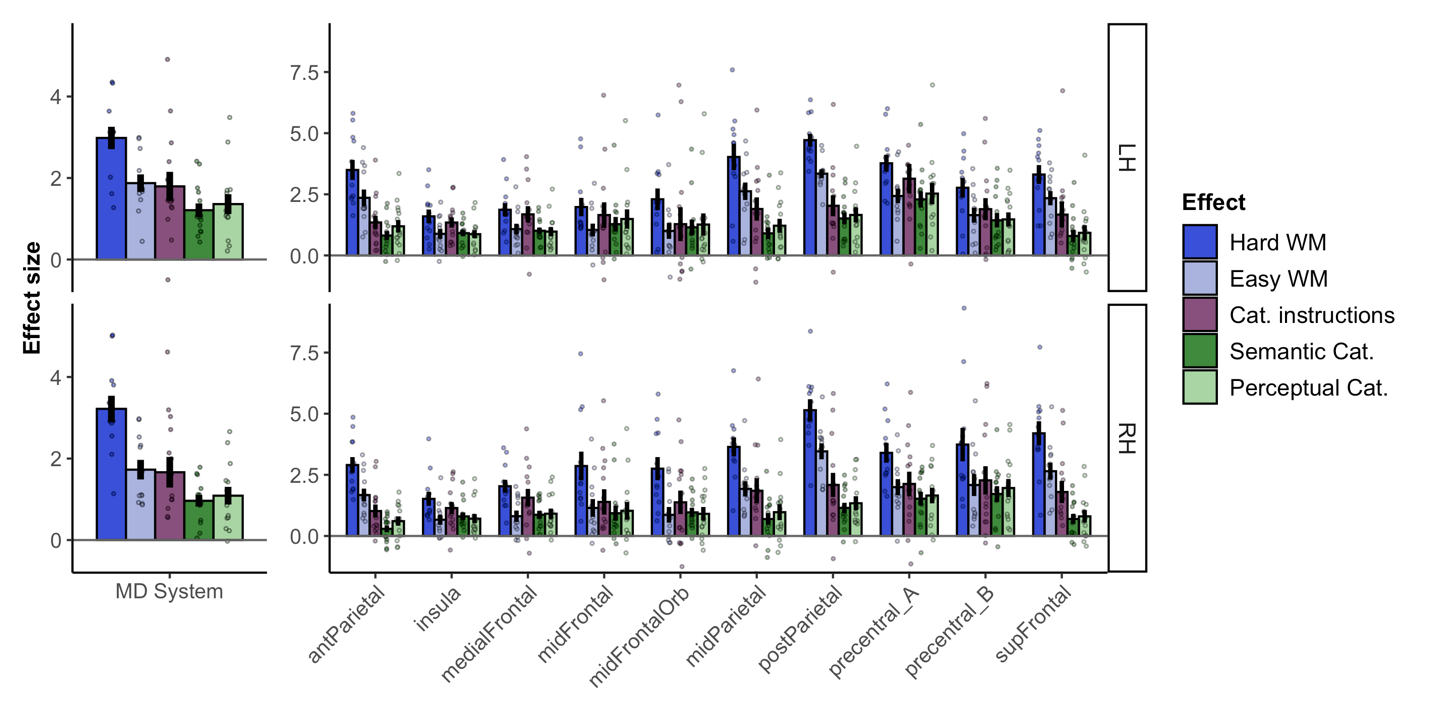


***Figure 2****. Categorization responses within the multiple demand brain network. Top: left hemisphere (LH); bottom: right hemisphere (RH). Left: responses averaged across fROIs; right: responses in individual fROIs. Abbreviations: WM – working memory task, Cat. – categorization, ant – anterior, mid – middle, post – posterior, precentral_A – dorsal precentral gyrus, precentral_B – ventral precentral gyrus.*

Finally, we conducted a whole-brain analysis to identify fROIs that might respond more strongly to semantic or perceptual categorization but lie outside the language and multiple demand fROIs described above. The GSS analysis revealed no regions that passed the threshold of having significant voxels in at least 60% of participants, indicating that no brain region exhibits a consistent preference for either semantic or perceptual categorization (as defined in our study).

**Table 3**. Mixed-effect linear regression results for individual fROIs within the language network. P values are FDR-corrected for the number of regions (n=12). SEM – semantic categorization, PERC – perceptual categorization, Instruct – instruction/category label processing, S – sentence reading (language localizer), N – nonword reading (language localizer).

| **fROI** | **Hemisphere** | **Regression Term** | **Beta** | **p value** | |
| --- | --- | --- | --- | --- | --- |
| IFG_orb | LH | Intercept | 0.48 | 0.031 | * |
|  |  | PERC-SEM | -0.08 | 0.866 |  |
|  |  | S-SEM | 1.17 | <.001 | *** |
|  |  | N-SEM | 0 | 0.99 |  |
|  |  | Instruct-SEM | 0.94 | <.001 | *** |
| IFG | LH | Intercept | 0.94 | <.001 | *** |
|  |  | PERC-SEM | -0.11 | 0.866 |  |
|  |  | S-SEM | 1.32 | <.001 | *** |
|  |  | N-SEM | -0.25 | 0.505 |  |
|  |  | Instruct-SEM | 1.51 | <.001 | *** |
| MFG | LH | Intercept | 0.64 | 0.031 | * |
|  |  | PERC-SEM | 0.1 | 0.866 |  |
|  |  | S-SEM | 2.38 | <.001 | *** |
|  |  | N-SEM | 0.76 | 0.248 |  |
|  |  | Instruct-SEM | 1.66 | <.001 | *** |
| AntTemp | LH | Intercept | -0.04 | 0.68 |  |
|  |  | PERC-SEM | -0.11 | 0.866 |  |
|  |  | S-SEM | 1.49 | <.001 | *** |
|  |  | N-SEM | 0.18 | 0.505 |  |
|  |  | Instruct-SEM | 1.09 | <.001 | *** |
| PostTemp | LH | Intercept | 0.31 | 0.204 |  |
|  |  | PERC-SEM | 0.06 | 0.866 |  |
|  |  | S-SEM | 1.82 | <.001 | *** |
|  |  | N-SEM | 0.26 | 0.505 |  |
|  |  | Instruct-SEM | 2.1 | <.001 | *** |
| AngG | LH | Intercept | 0.21 | 0.416 |  |
|  |  | PERC-SEM | -0.44 | 0.866 |  |
|  |  | S-SEM | 0.34 | 0.207 |  |
|  |  | N-SEM | -0.54 | 0.248 |  |
|  |  | Instruct-SEM | 0.47 | 0.083 |  |
| IFG_orb | RH | Intercept | 0.18 | 0.416 |  |
|  |  | PERC-SEM | -0.12 | 0.866 |  |
|  |  | S-SEM | 0.59 | 0.016 | * |
|  |  | N-SEM | 0.15 | 0.686 |  |
|  |  | Instruct-SEM | 0.23 | 0.313 |  |
| IFG | RH | Intercept | 0.33 | 0.126 |  |
|  |  | PERC-SEM | -0.13 | 0.866 |  |
|  |  | S-SEM | 0.48 | 0.034 | * |
|  |  | N-SEM | -0.1 | 0.765 |  |
|  |  | Instruct-SEM | 0.53 | 0.019 | * |
| MFG | RH | Intercept | 0.57 | 0.039 | * |
|  |  | PERC-SEM | 0.08 | 0.866 |  |
|  |  | S-SEM | 0.3 | 0.177 |  |
|  |  | N-SEM | 0 | 0.99 |  |
|  |  | Instruct-SEM | 0.73 | 0.001 | ** |
| AntTemp | RH | Intercept | -0.18 | 0.392 |  |
|  |  | PERC-SEM | -0.15 | 0.866 |  |
|  |  | S-SEM | 1.21 | <.001 | *** |
|  |  | N-SEM | 0.3 | 0.482 |  |
|  |  | Instruct-SEM | 1.02 | <.001 | *** |
| PostTemp | RH | Intercept | 0.07 | 0.68 |  |
|  |  | PERC-SEM | -0.02 | 0.927 |  |
|  |  | S-SEM | 1.1 | <.001 | *** |
|  |  | N-SEM | 0.35 | 0.248 |  |
|  |  | Instruct-SEM | 1.37 | <.001 | *** |
| AngG | RH | Intercept | -0.09 | 0.68 |  |
|  |  | PERC-SEM | -0.25 | 0.866 |  |
|  |  | S-SEM | -0.04 | 0.886 |  |
|  |  | N-SEM | -0.19 | 0.686 |  |
|  |  | Instruct-SEM | 0.58 | 0.027 | * |

**Table 4**. Mixed-effect linear regression results for individual fROIs within the multiple demand system. P values are FDR-corrected for the number of regions (n=20). SEM – semantic categorization, PERC – perceptual categorization, Instruct – instruction/category label processing, S – sentence reading (language localizer), N – nonword reading (language localizer), H – hard working memory task (multiple demand localizer), E – easy working memory task (multiple demand localizer).

| **fROI** | **Hemisphere** | **Regression Term** | **Beta** | **p value** | |
| --- | --- | --- | --- | --- | --- |
| postParietal | LH | Intercept | 1.59 | <.001 | *** |
|  |  | PERC-SEM | 0.14 | 0.901 |  |
|  |  | H-E | 1.37 | <.001 | *** |
|  |  | HE-SEM | 2.62 | <.001 | *** |
|  |  | N-SEM | -0.98 | 0.002 | ** |
|  |  | S-SEM | -1.43 | <.001 | *** |
|  |  | Instruct-SEM | 0.52 | 0.056 |  |
| midParietal | LH | Intercept | 1.06 | 0.003 | ** |
|  |  | PERC-SEM | 0.31 | 0.901 |  |
|  |  | H-E | 1.4 | 0.001 | ** |
|  |  | HE-SEM | 2.38 | <.001 | *** |
|  |  | N-SEM | 0.02 | 0.96 |  |
|  |  | S-SEM | -0.57 | 0.169 |  |
|  |  | Instruct-SEM | 1 | 0.025 | * |
| antParietal | LH | Intercept | 1 | <.001 | *** |
|  |  | PERC-SEM | 0.38 | 0.901 |  |
|  |  | H-E | 1.14 | <.001 | *** |
|  |  | HE-SEM | 2.17 | <.001 | *** |
|  |  | N-SEM | -0.1 | 0.873 |  |
|  |  | S-SEM | -0.64 | 0.023 | * |
|  |  | Instruct-SEM | 0.54 | 0.064 |  |
| supFrontal | LH | Intercept | 0.86 | 0.009 | ** |
|  |  | PERC-SEM | 0.13 | 0.901 |  |
|  |  | H-E | 0.97 | 0.007 | ** |
|  |  | HE-SEM | 2.11 | <.001 | *** |
|  |  | N-SEM | -0.28 | 0.55 |  |
|  |  | S-SEM | -0.4 | 0.256 |  |
|  |  | Instruct-SEM | 0.88 | 0.025 | * |
| precentral_A | LH | Intercept | 2.41 | <.001 | *** |
|  |  | PERC-SEM | 0.24 | 0.901 |  |
|  |  | H-E | 1.34 | <.001 | *** |
|  |  | HE-SEM | 0.94 | 0.003 | ** |
|  |  | N-SEM | -0.62 | 0.155 |  |
|  |  | S-SEM | -1.03 | 0.008 | ** |
|  |  | Instruct-SEM | 0.85 | 0.025 | * |
| precentral_B | LH | Intercept | 1.46 | <.001 | *** |
|  |  | PERC-SEM | 0.04 | 0.901 |  |
|  |  | H-E | 1.12 | 0.001 | ** |
|  |  | HE-SEM | 0.67 | 0.017 | * |
|  |  | N-SEM | -0.68 | 0.091 |  |
|  |  | S-SEM | -0.79 | 0.021 | * |
|  |  | Instruct-SEM | 0.45 | 0.18 |  |
| midFrontal | LH | Intercept | 1.38 | <.001 | *** |
|  |  | PERC-SEM | 0.21 | 0.901 |  |
|  |  | H-E | 0.94 | 0.003 | ** |
|  |  | HE-SEM | 0.27 | 0.299 |  |
|  |  | N-SEM | -0.83 | 0.032 | * |
|  |  | S-SEM | -1.21 | <.001 | *** |
|  |  | Instruct-SEM | 0.38 | 0.216 |  |
| midFrontalOrb | LH | Intercept | 1.21 | 0.009 | ** |
|  |  | PERC-SEM | 0.12 | 0.901 |  |
|  |  | H-E | 1.29 | 0.002 | ** |
|  |  | HE-SEM | 0.67 | 0.052 |  |
|  |  | N-SEM | -0.58 | 0.225 |  |
|  |  | S-SEM | -1.04 | 0.016 | * |
|  |  | Instruct-SEM | 0.13 | 0.727 |  |
| insula | LH | Intercept | 0.89 | <.001 | *** |
|  |  | PERC-SEM | -0.04 | 0.901 |  |
|  |  | H-E | 0.72 | <.001 | *** |
|  |  | HE-SEM | 0.38 | 0.006 | ** |
|  |  | N-SEM | -0.45 | 0.021 | * |
|  |  | S-SEM | -0.57 | <.001 | *** |
|  |  | Instruct-SEM | 0.43 | 0.016 | * |
| medialFrontal | LH | Intercept | 0.99 | <.001 | *** |
|  |  | PERC-SEM | -0.03 | 0.901 |  |
|  |  | H-E | 0.79 | 0.001 | ** |
|  |  | HE-SEM | 0.51 | 0.012 | * |
|  |  | N-SEM | -0.43 | 0.124 |  |
|  |  | S-SEM | -0.6 | 0.016 | * |
|  |  | Instruct-SEM | 0.67 | 0.016 | * |
| postParietal | RH | Intercept | 1.24 | <.001 | *** |
|  |  | PERC-SEM | 0.19 | 0.901 |  |
|  |  | H-E | 1.68 | <.001 | *** |
|  |  | HE-SEM | 3.18 | <.001 | *** |
|  |  | N-SEM | -0.84 | 0.068 |  |
|  |  | S-SEM | -1.14 | 0.006 | ** |
|  |  | Instruct-SEM | 0.94 | 0.025 | * |
| midParietal | RH | Intercept | 0.83 | 0.006 | ** |
|  |  | PERC-SEM | 0.28 | 0.901 |  |
|  |  | H-E | 1.72 | <.001 | *** |
|  |  | HE-SEM | 2.14 | <.001 | *** |
|  |  | N-SEM | 0.12 | 0.873 |  |
|  |  | S-SEM | -0.36 | 0.375 |  |
|  |  | Instruct-SEM | 1.16 | 0.016 | * |
| antParietal | RH | Intercept | 0.45 | 0.009 | ** |
|  |  | PERC-SEM | 0.32 | 0.901 |  |
|  |  | H-E | 1.23 | <.001 | *** |
|  |  | HE-SEM | 2.02 | <.001 | *** |
|  |  | N-SEM | 0.06 | 0.928 |  |
|  |  | S-SEM | -0.2 | 0.515 |  |
|  |  | Instruct-SEM | 0.73 | 0.029 | * |
| supFrontal | RH | Intercept | 0.75 | 0.015 | * |
|  |  | PERC-SEM | 0.11 | 0.901 |  |
|  |  | H-E | 1.55 | <.001 | *** |
|  |  | HE-SEM | 2.76 | <.001 | *** |
|  |  | N-SEM | -0.05 | 0.948 |  |
|  |  | S-SEM | -0.2 | 0.594 |  |
|  |  | Instruct-SEM | 1.1 | 0.019 | * |
| precentral_A | RH | Intercept | 1.59 | <.001 | *** |
|  |  | PERC-SEM | 0.13 | 0.901 |  |
|  |  | H-E | 1.4 | 0.001 | ** |
|  |  | HE-SEM | 1.18 | 0.002 | ** |
|  |  | N-SEM | -0.4 | 0.438 |  |
|  |  | S-SEM | -0.74 | 0.074 |  |
|  |  | Instruct-SEM | 0.6 | 0.157 |  |
| precentral_B | RH | Intercept | 1.84 | <.001 | *** |
|  |  | PERC-SEM | 0.24 | 0.901 |  |
|  |  | H-E | 1.65 | <.001 | *** |
|  |  | HE-SEM | 1.23 | 0.003 | ** |
|  |  | N-SEM | -0.68 | 0.225 |  |
|  |  | S-SEM | -1.06 | 0.023 | * |
|  |  | Instruct-SEM | 0.56 | 0.216 |  |
| midFrontal | RH | Intercept | 0.98 | 0.009 | ** |
|  |  | PERC-SEM | 0.1 | 0.901 |  |
|  |  | H-E | 1.71 | <.001 | *** |
|  |  | HE-SEM | 1.03 | 0.003 | ** |
|  |  | N-SEM | -0.24 | 0.662 |  |
|  |  | S-SEM | -0.63 | 0.105 |  |
|  |  | Instruct-SEM | 0.46 | 0.216 |  |
| midFrontalOrb | RH | Intercept | 0.94 | 0.004 | ** |
|  |  | PERC-SEM | -0.06 | 0.901 |  |
|  |  | H-E | 1.89 | <.001 | *** |
|  |  | HE-SEM | 0.87 | 0.012 | * |
|  |  | N-SEM | -0.42 | 0.404 |  |
|  |  | S-SEM | -0.91 | 0.023 | * |
|  |  | Instruct-SEM | 0.41 | 0.272 |  |
| insula | RH | Intercept | 0.75 | 0.001 | ** |
|  |  | PERC-SEM | -0.1 | 0.901 |  |
|  |  | H-E | 0.85 | <.001 | *** |
|  |  | HE-SEM | 0.32 | 0.019 | * |
|  |  | N-SEM | -0.39 | 0.048 | * |
|  |  | S-SEM | -0.52 | 0.003 | ** |
|  |  | Instruct-SEM | 0.34 | 0.04 | * |
| medialFrontal | RH | Intercept | 0.89 | <.001 | *** |
|  |  | PERC-SEM | 0.04 | 0.901 |  |
|  |  | H-E | 1.23 | <.001 | *** |
|  |  | HE-SEM | 0.6 | 0.006 | ** |
|  |  | N-SEM | -0.36 | 0.225 |  |
|  |  | S-SEM | -0.61 | 0.02 | * |
|  |  | Instruct-SEM | 0.7 | 0.016 | * |
